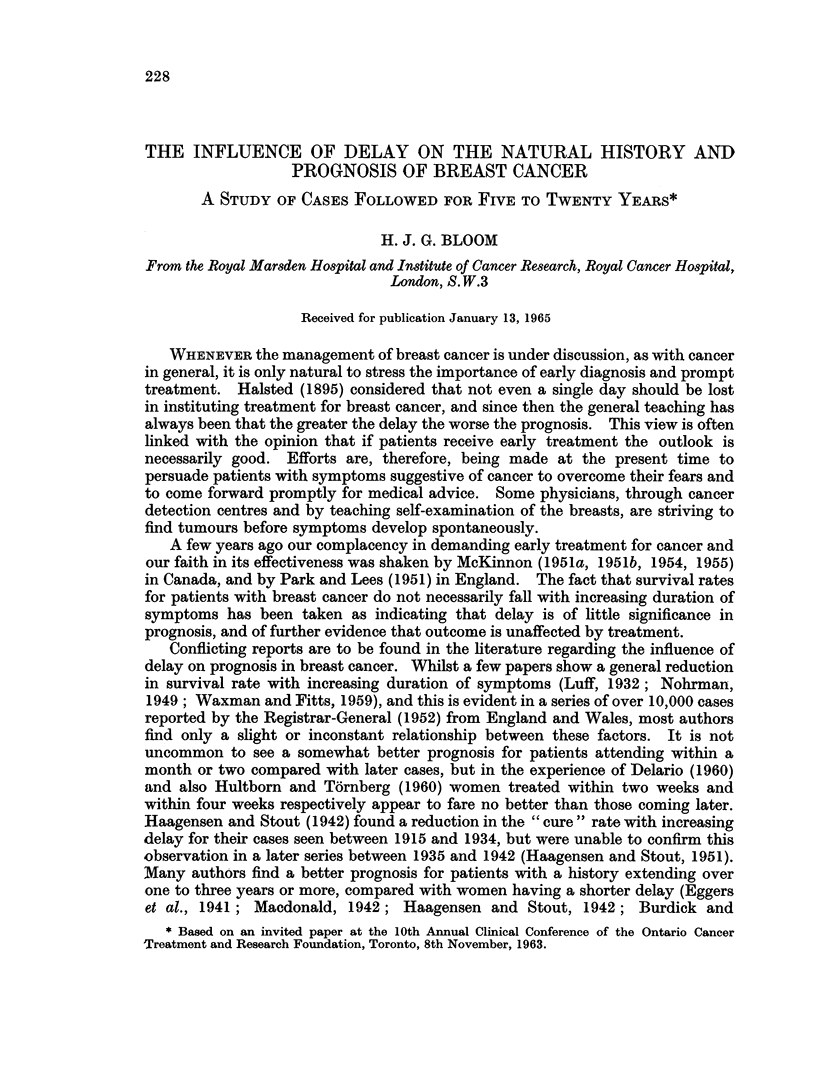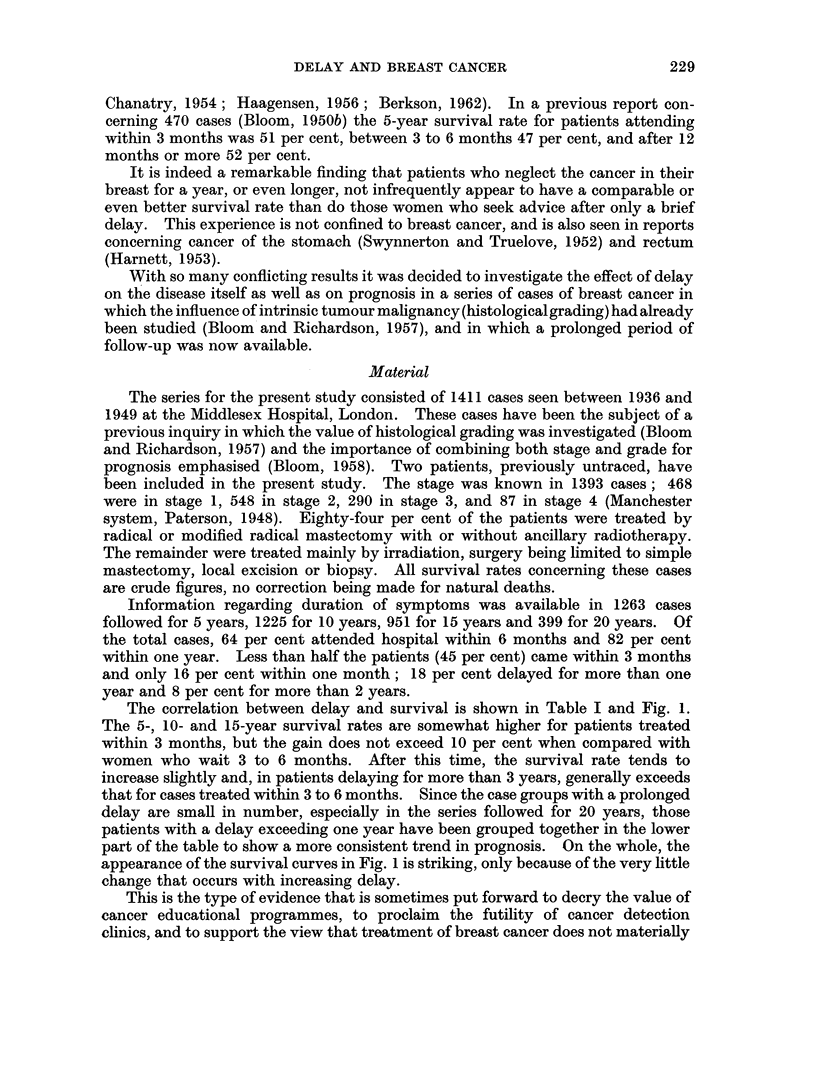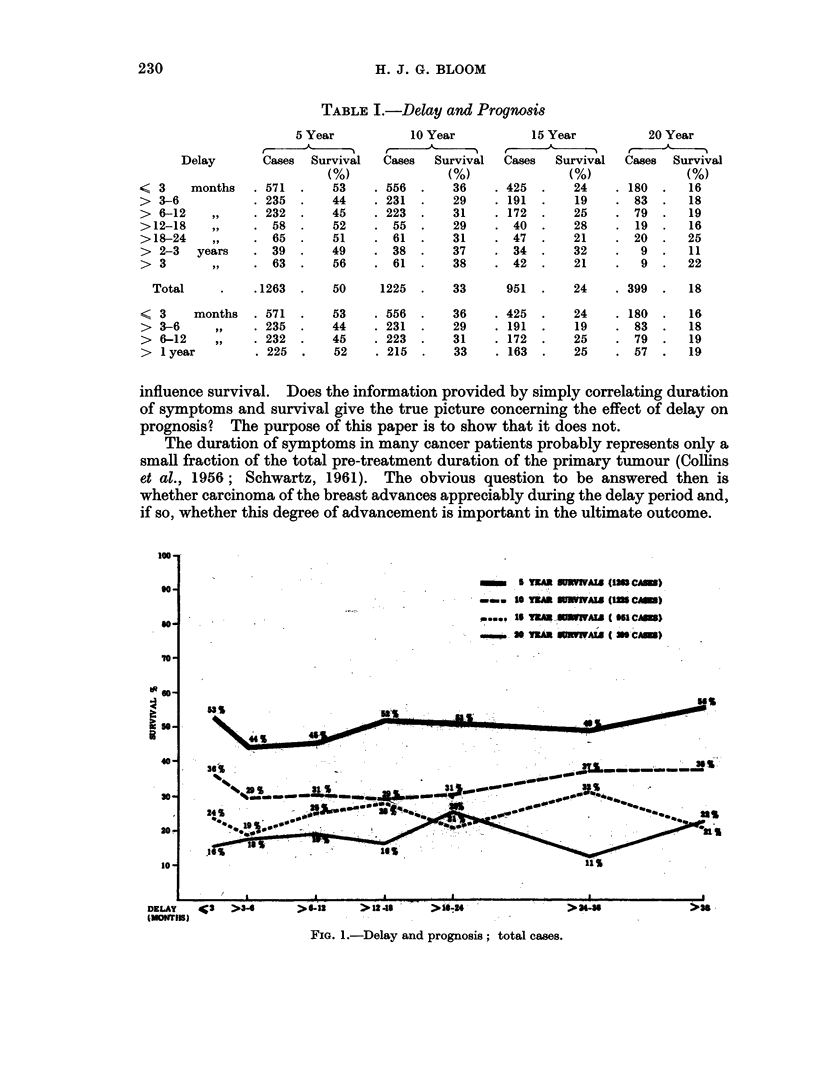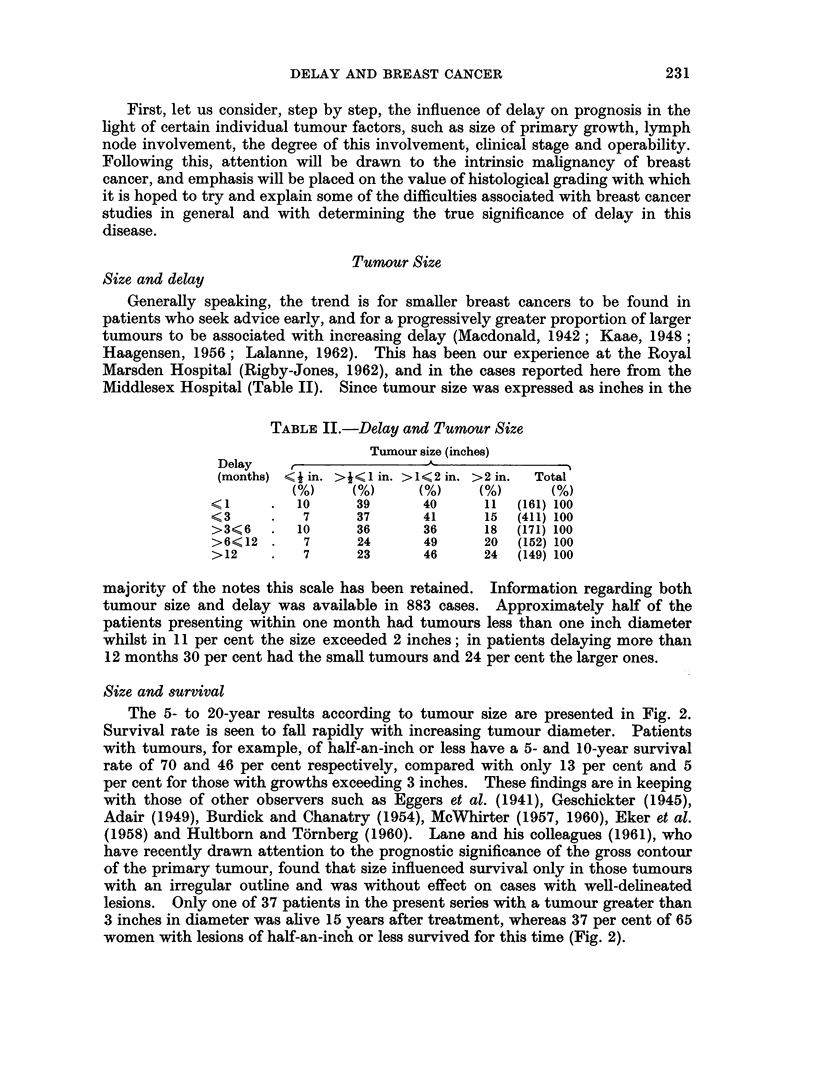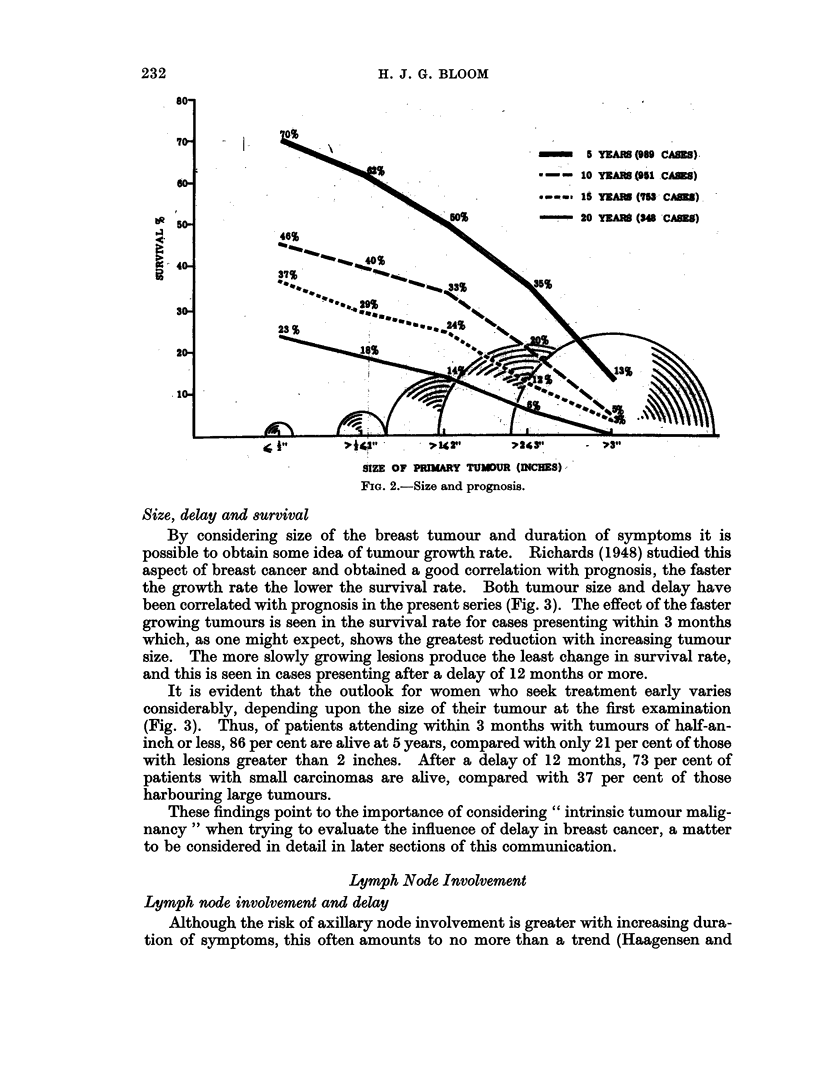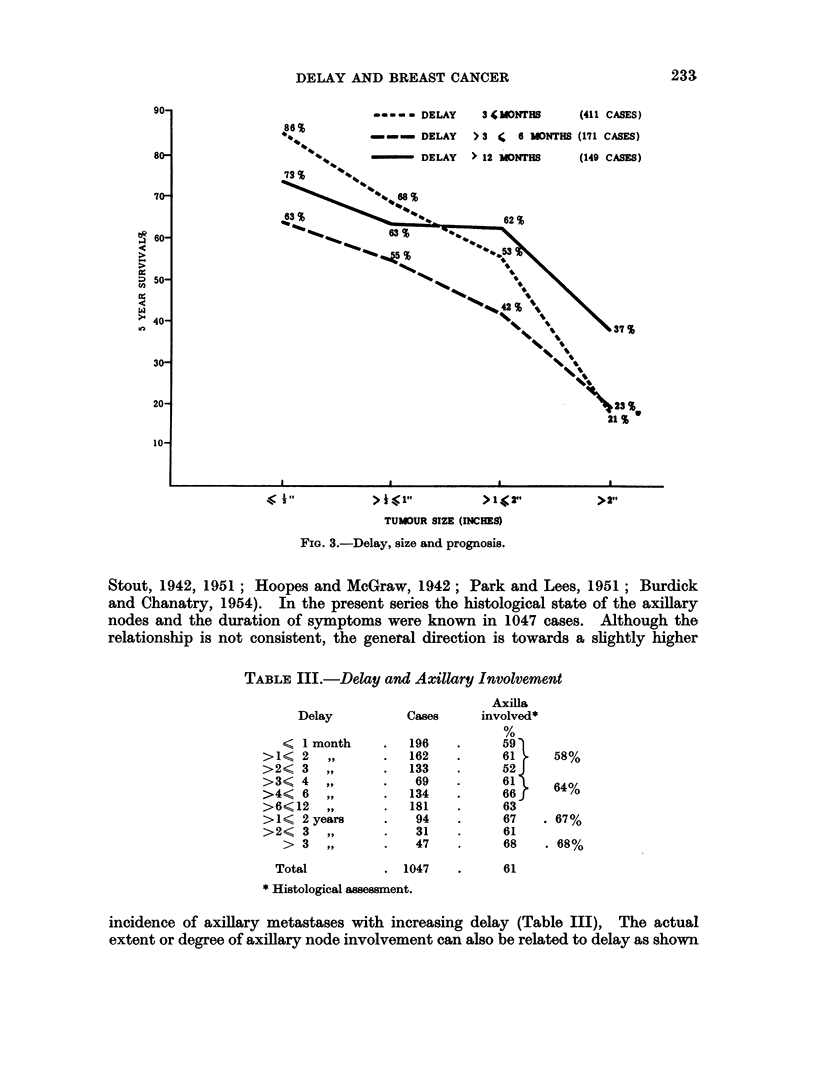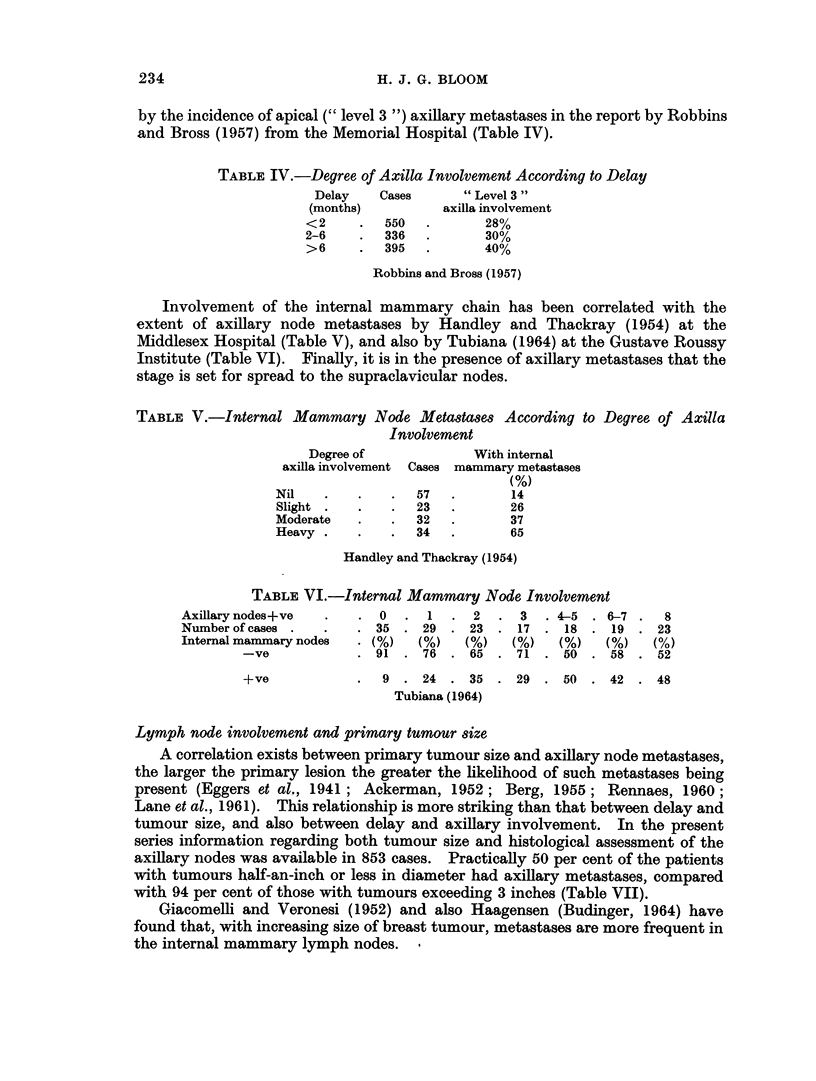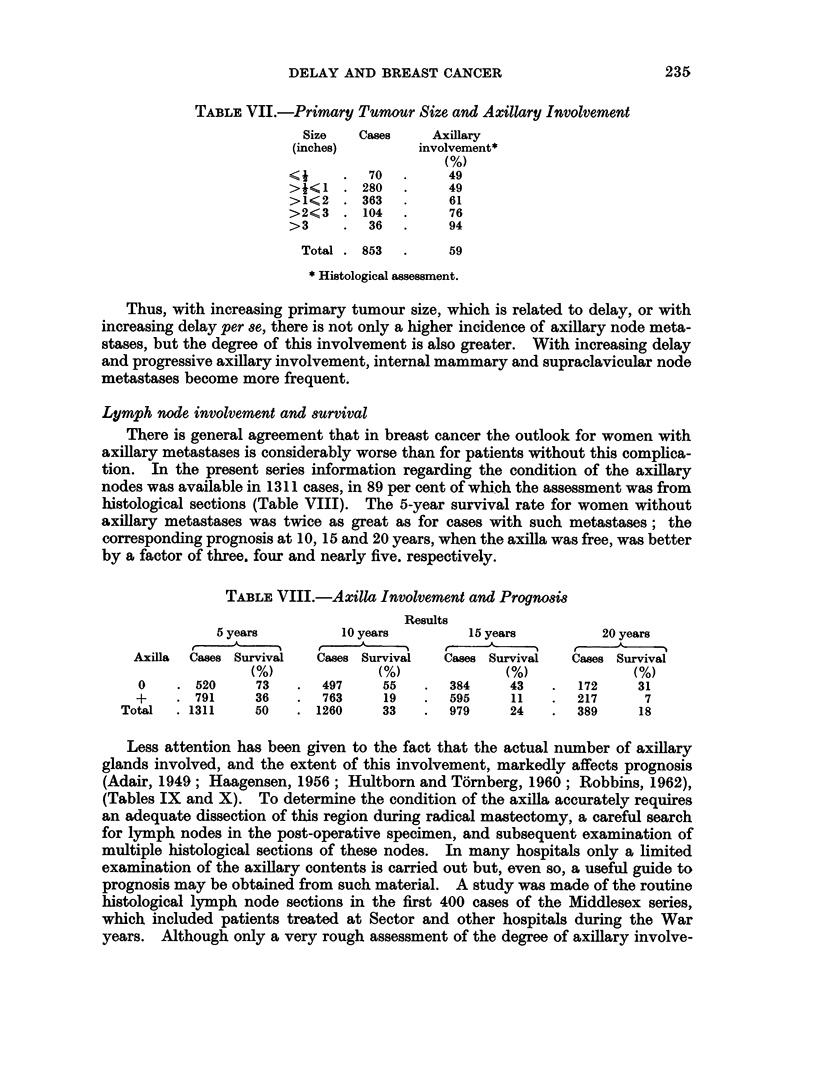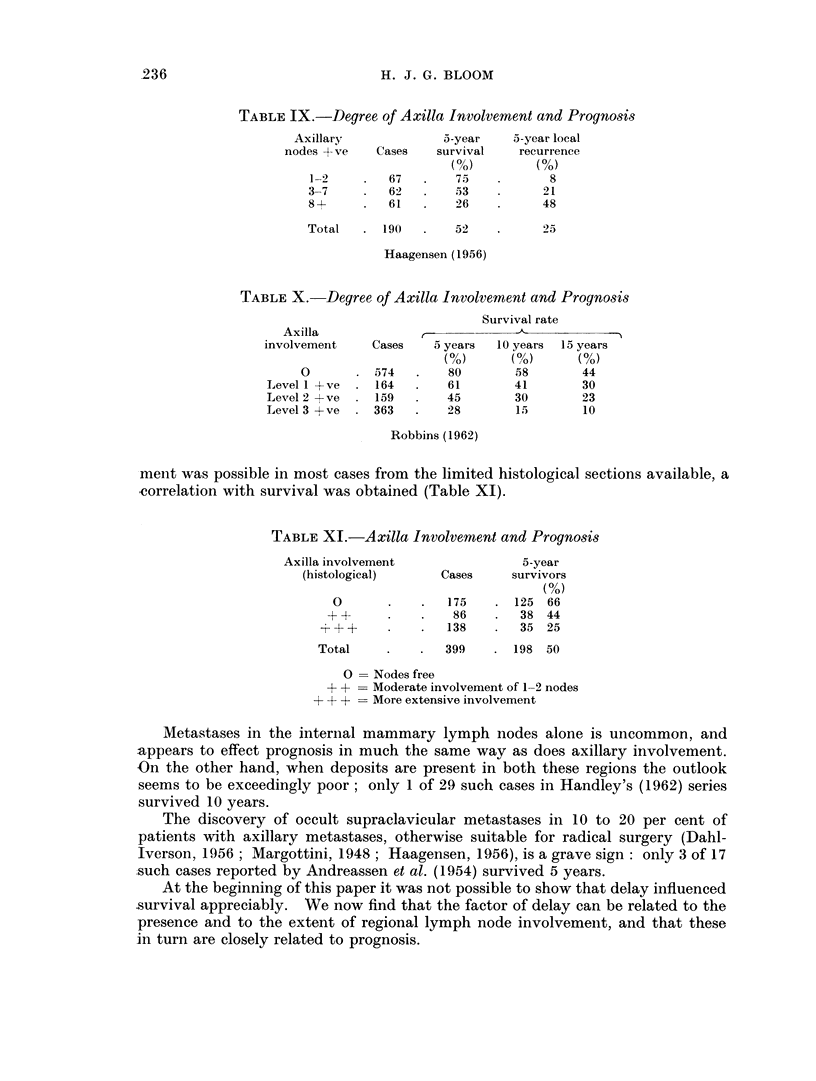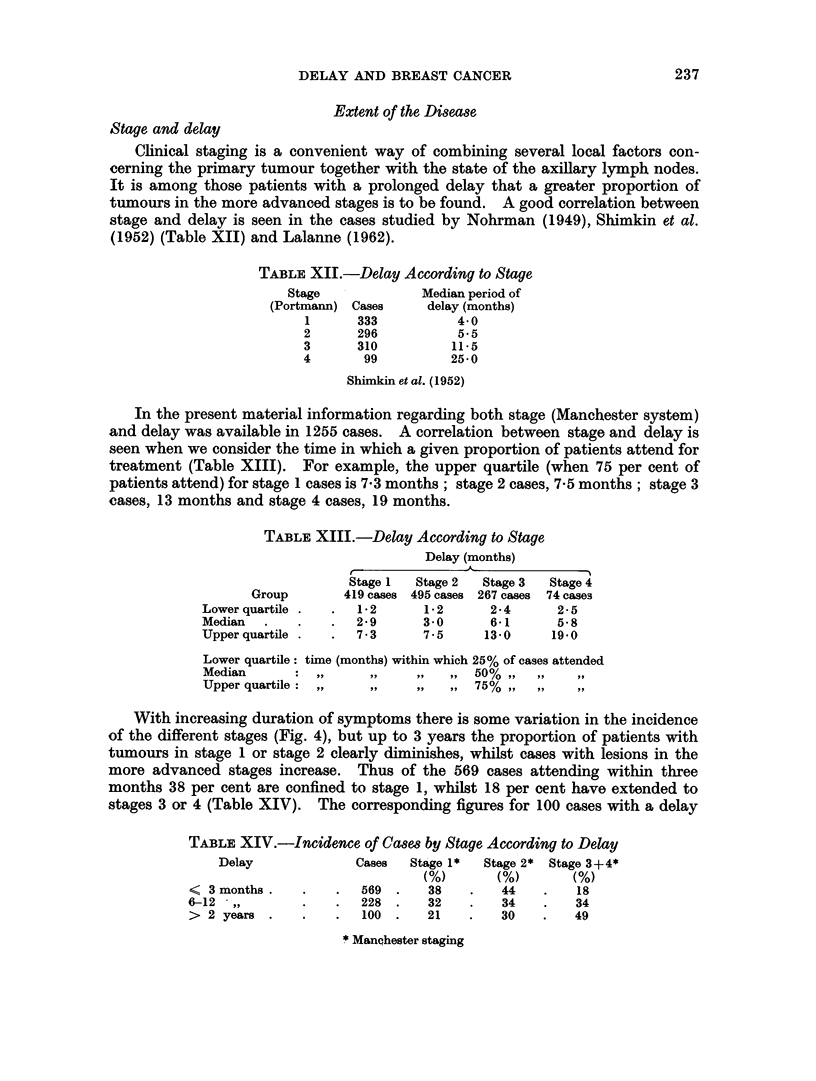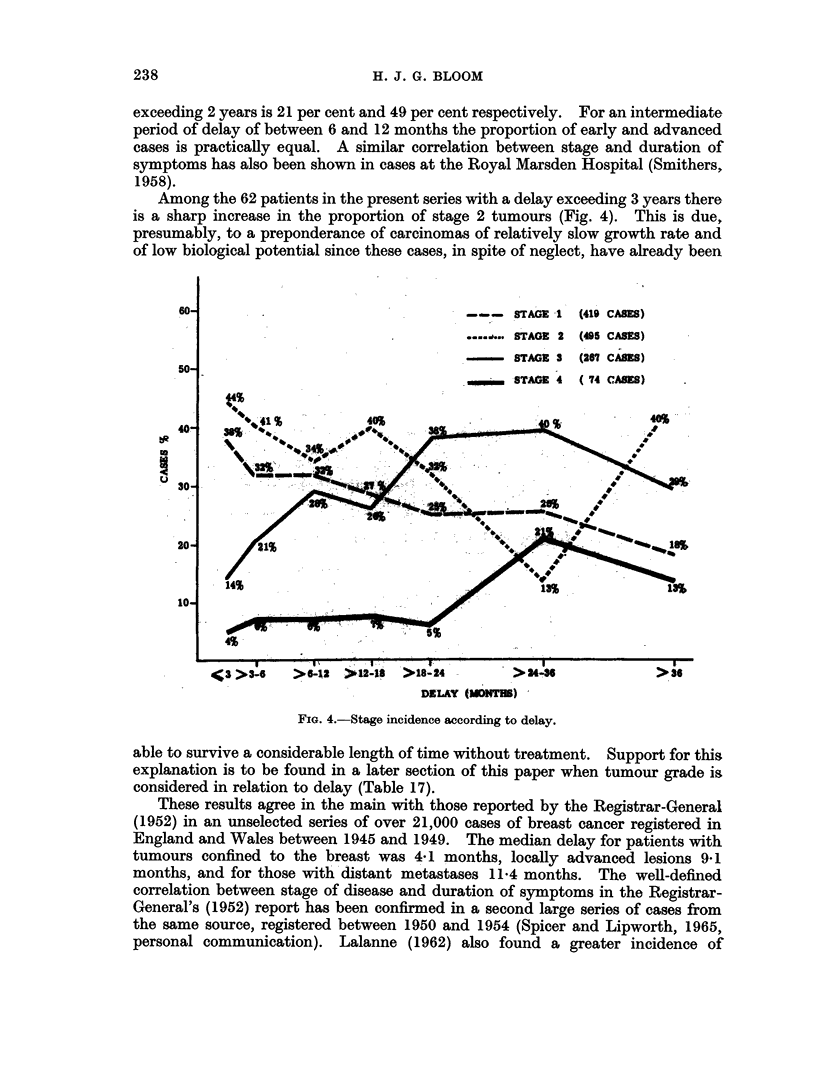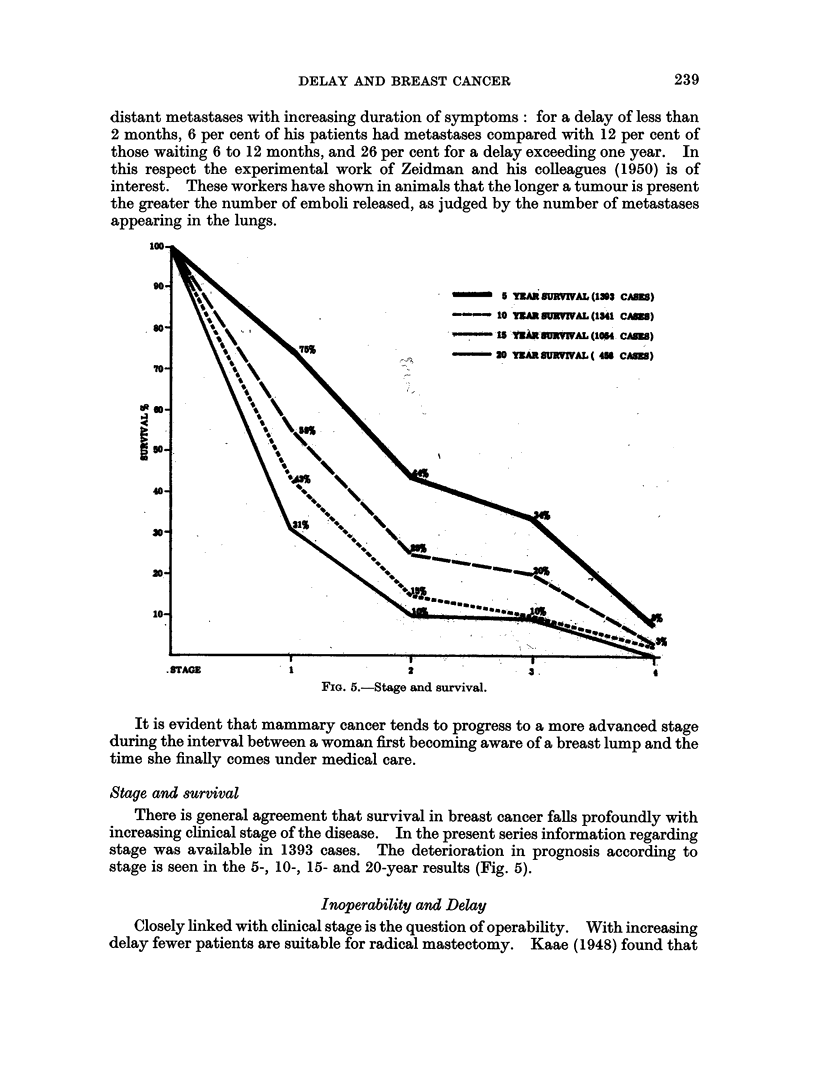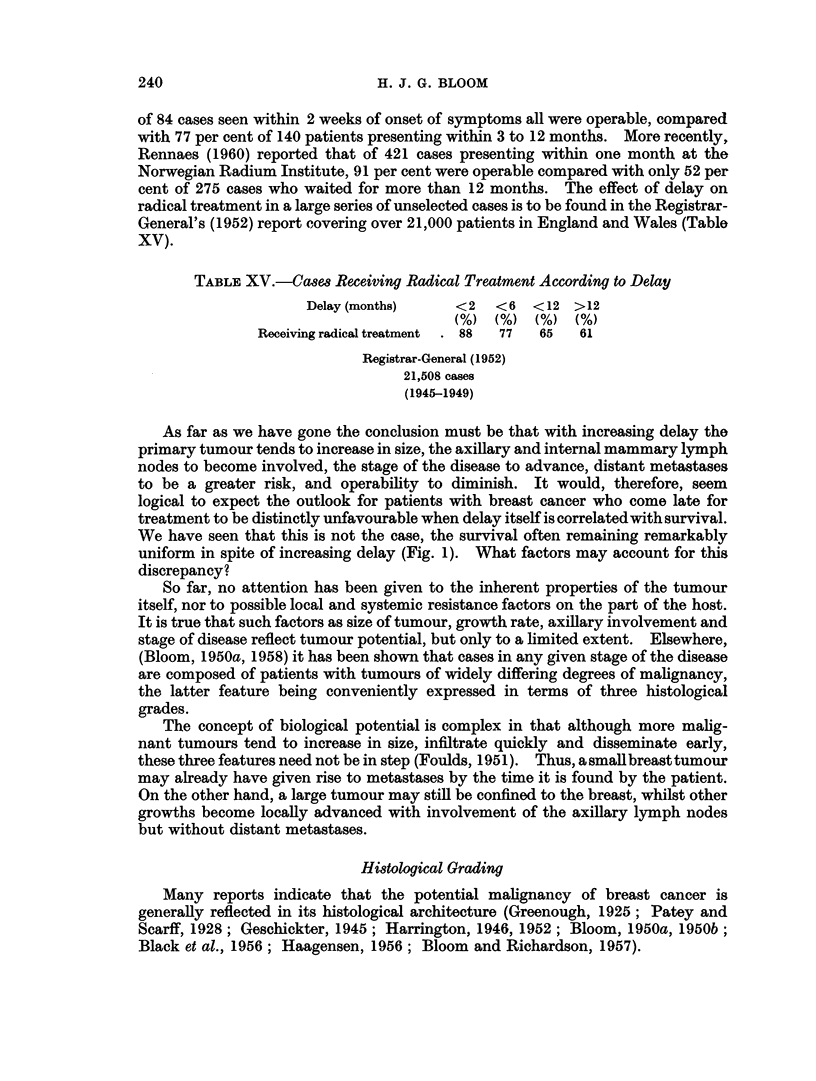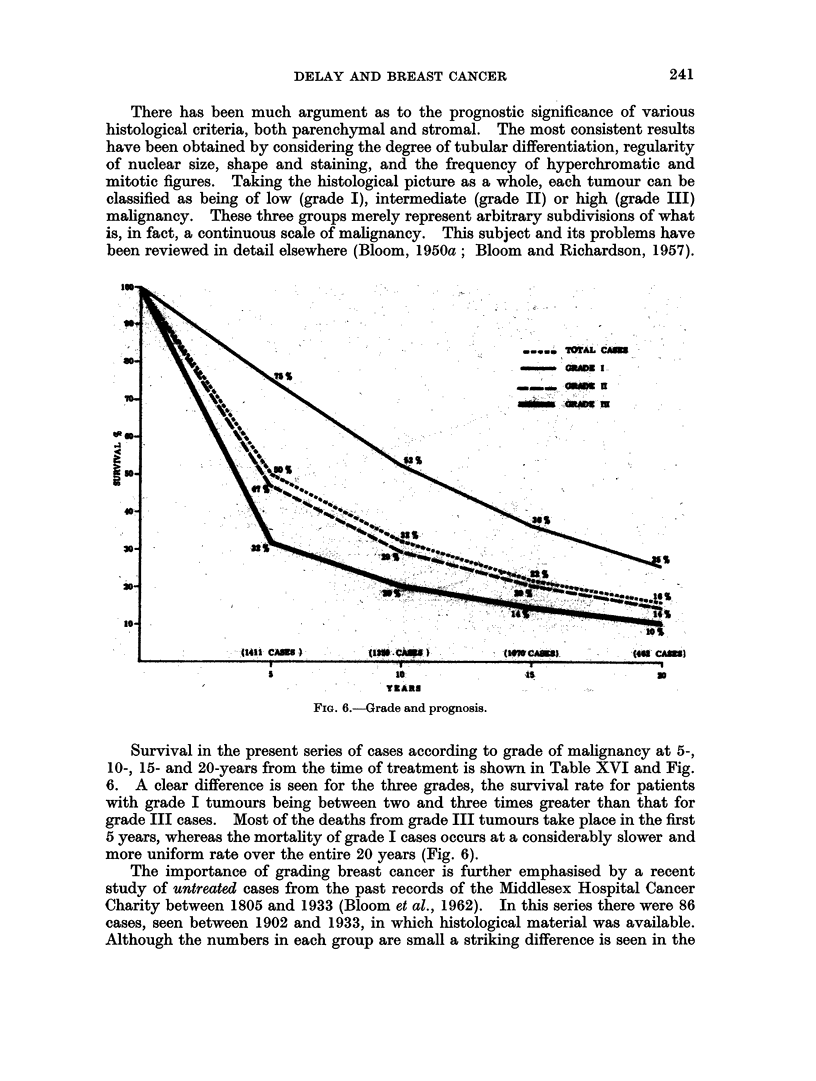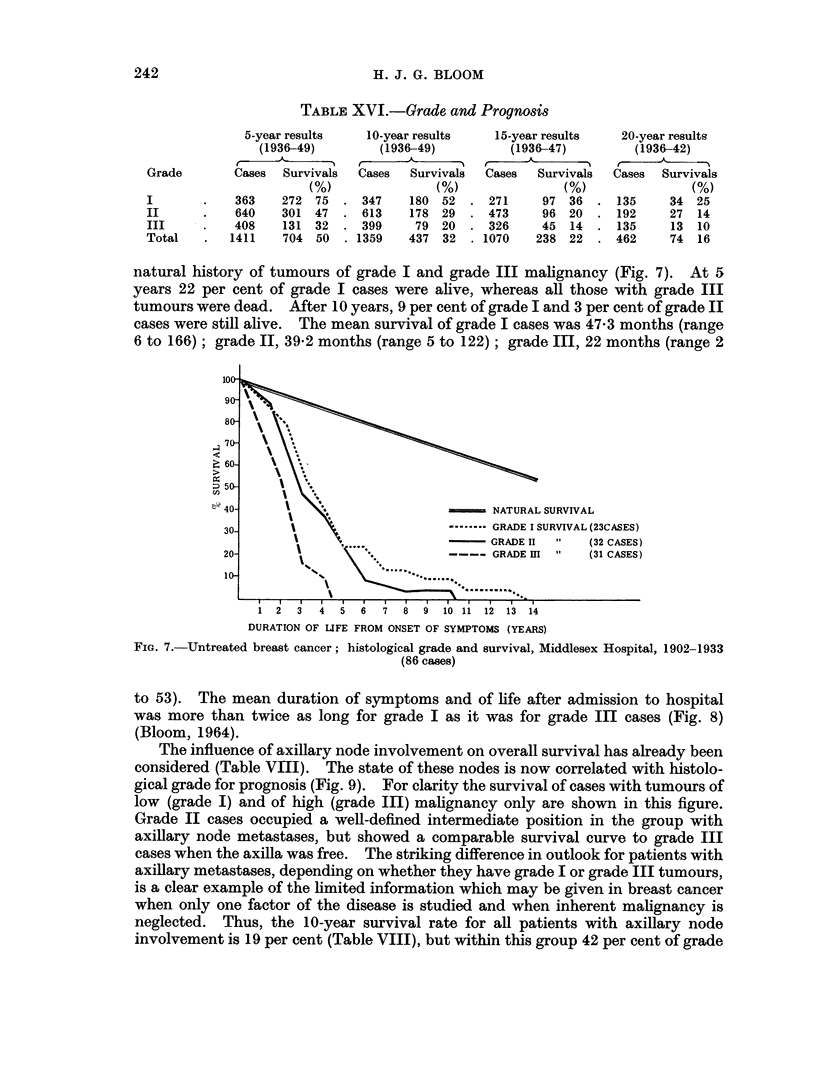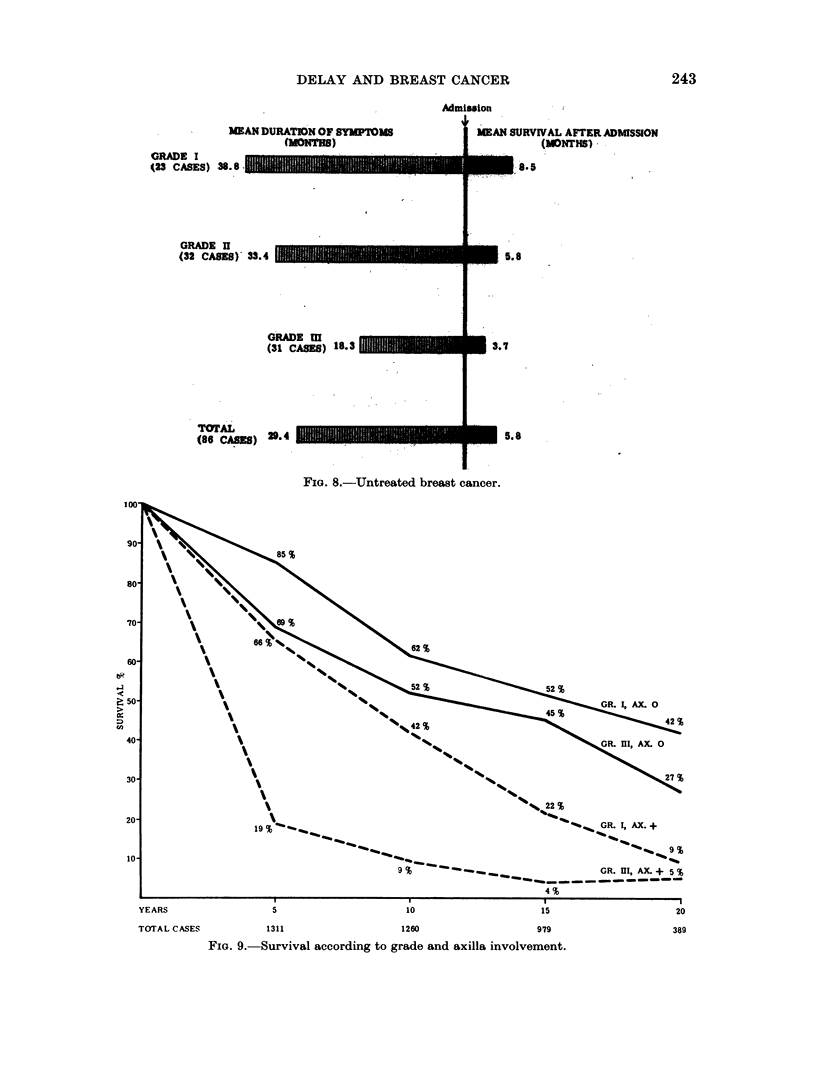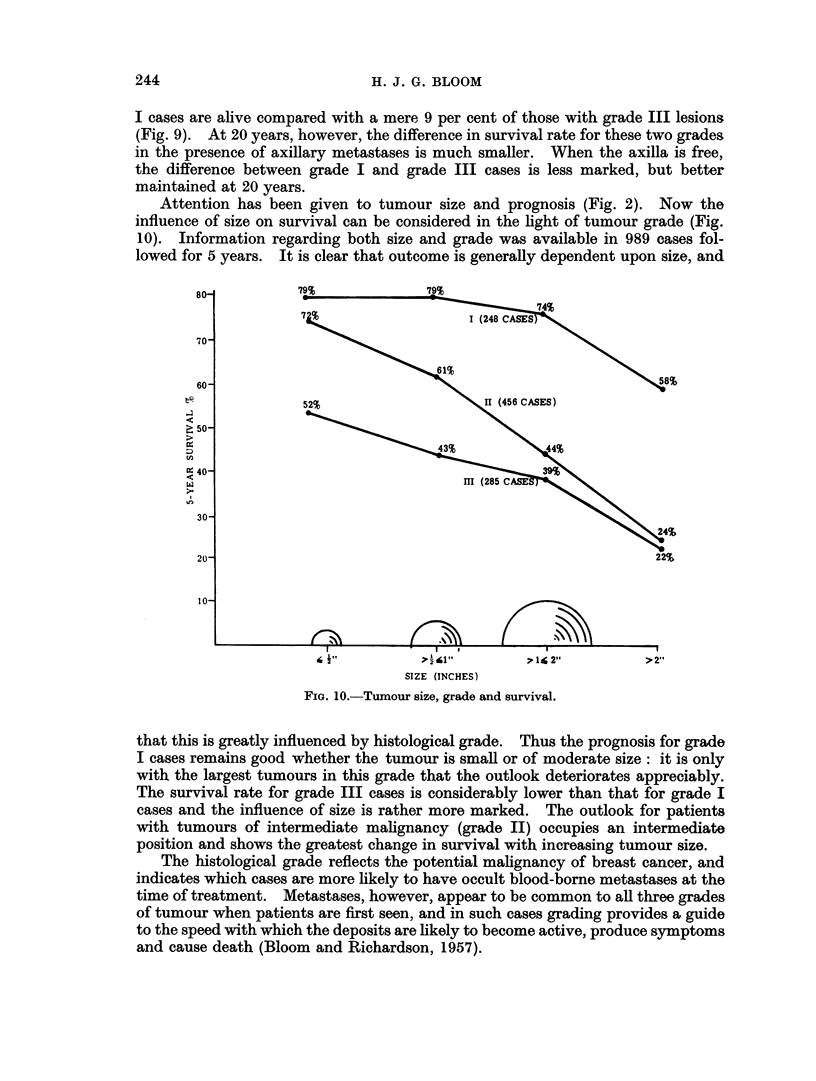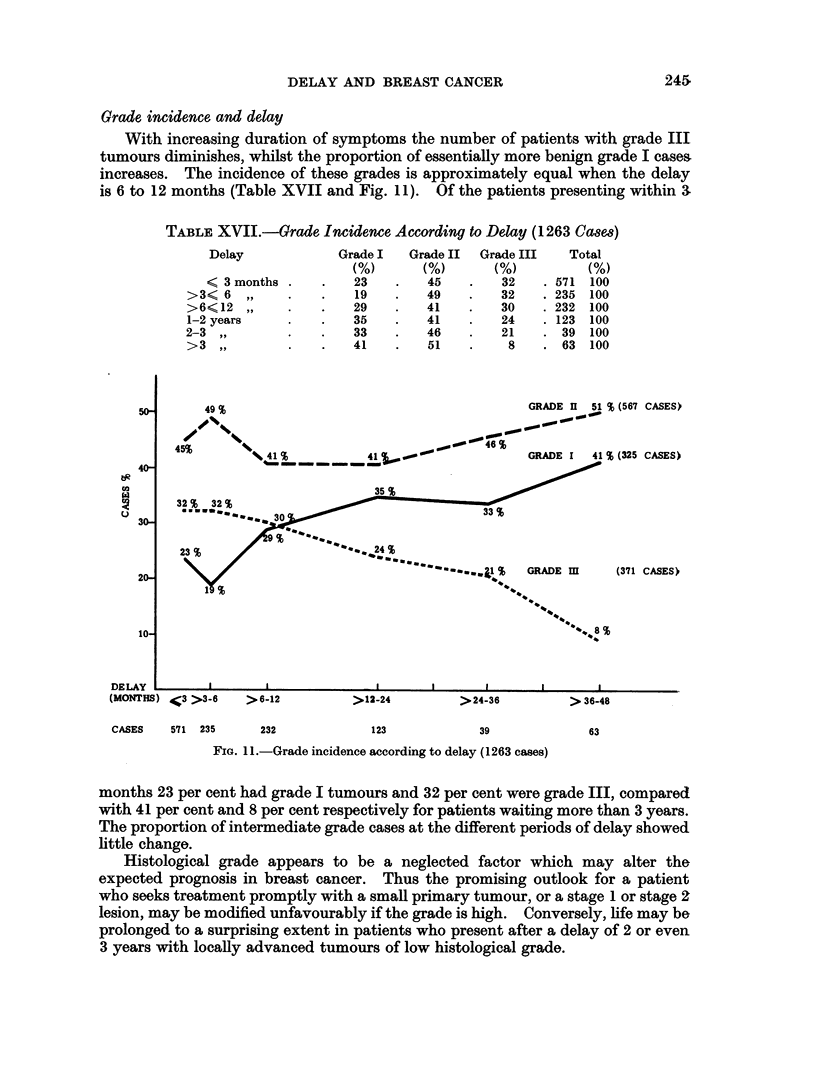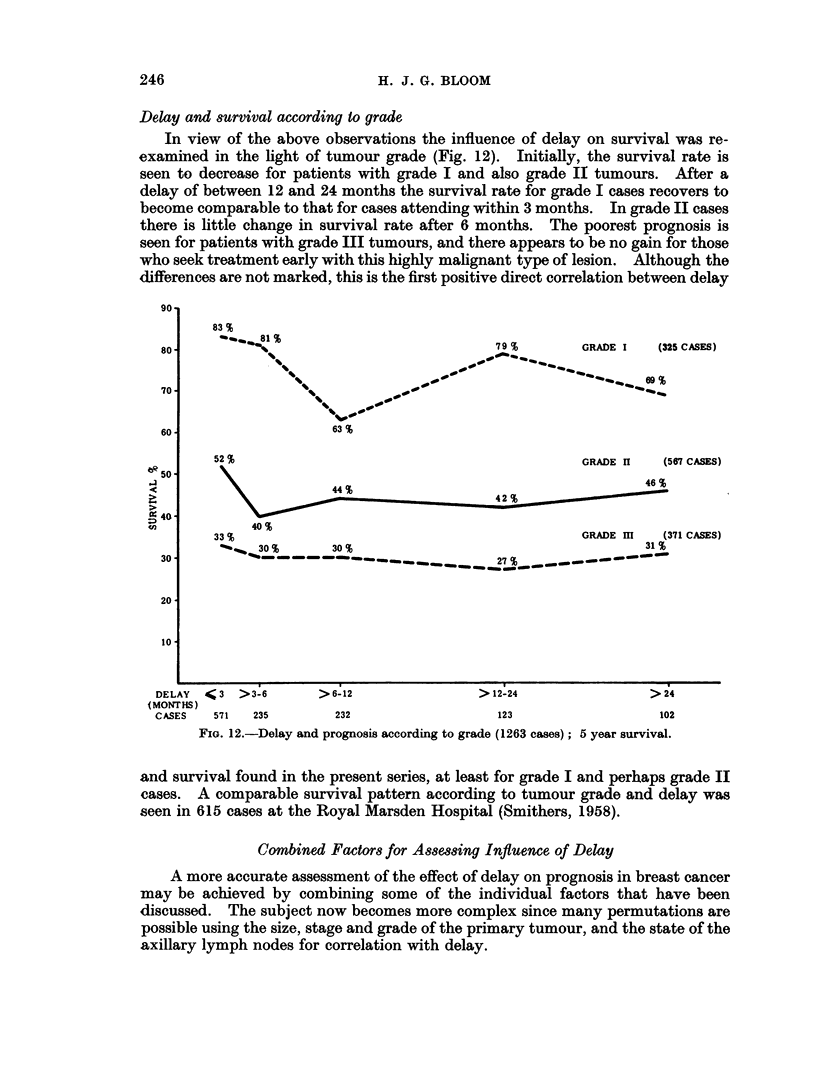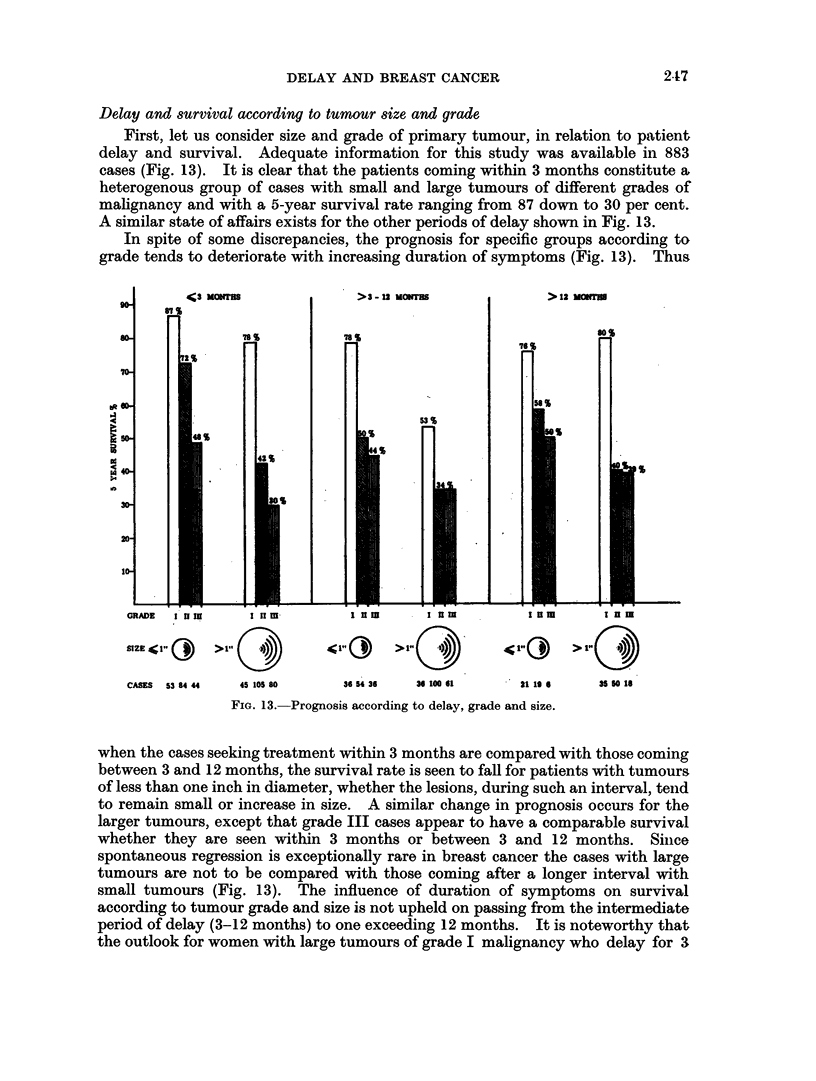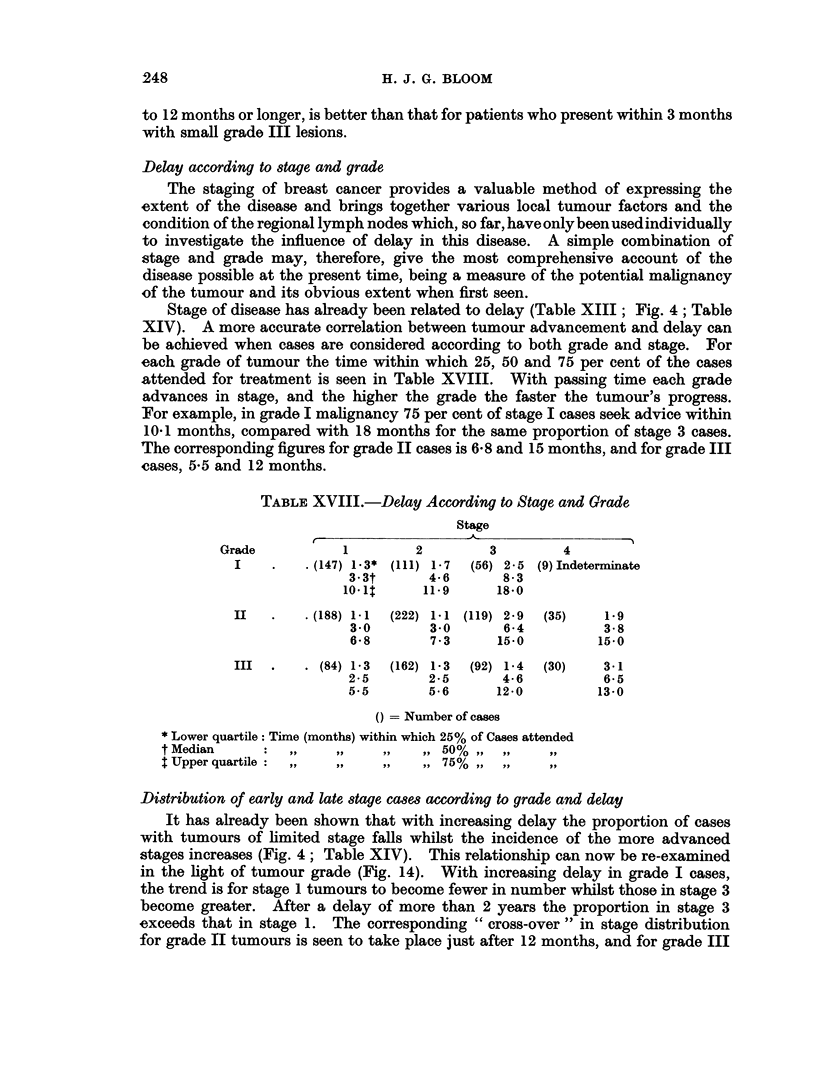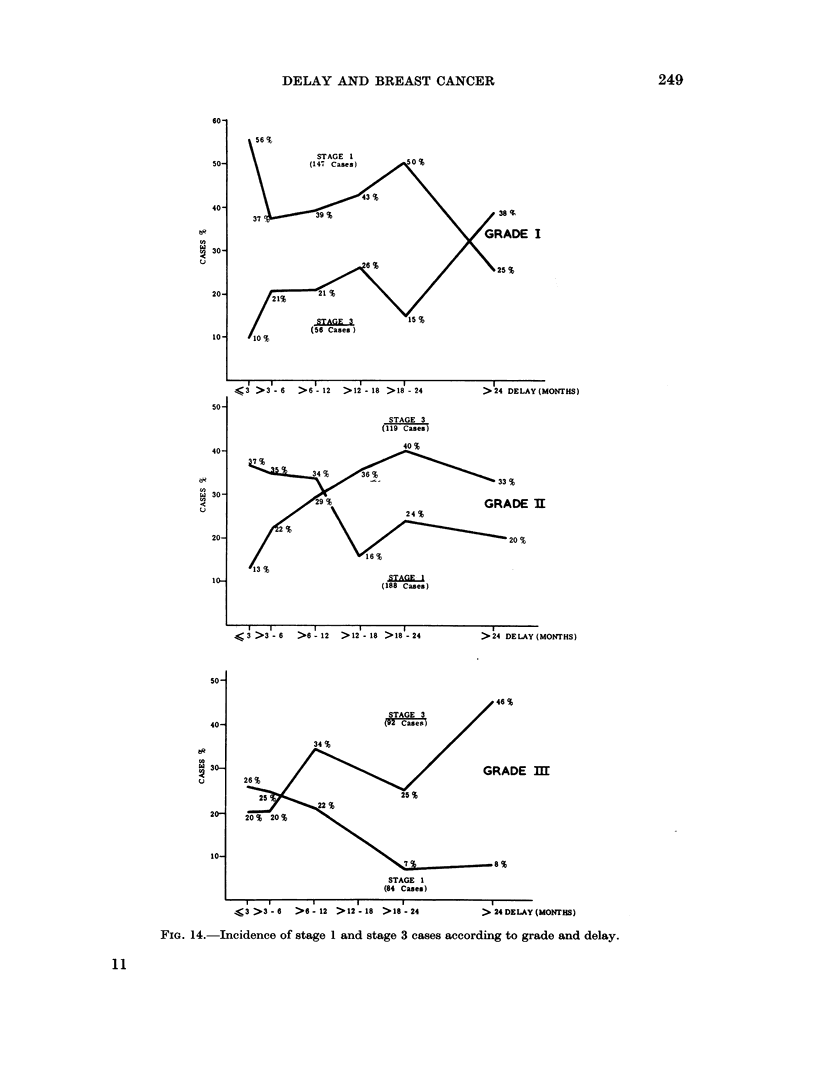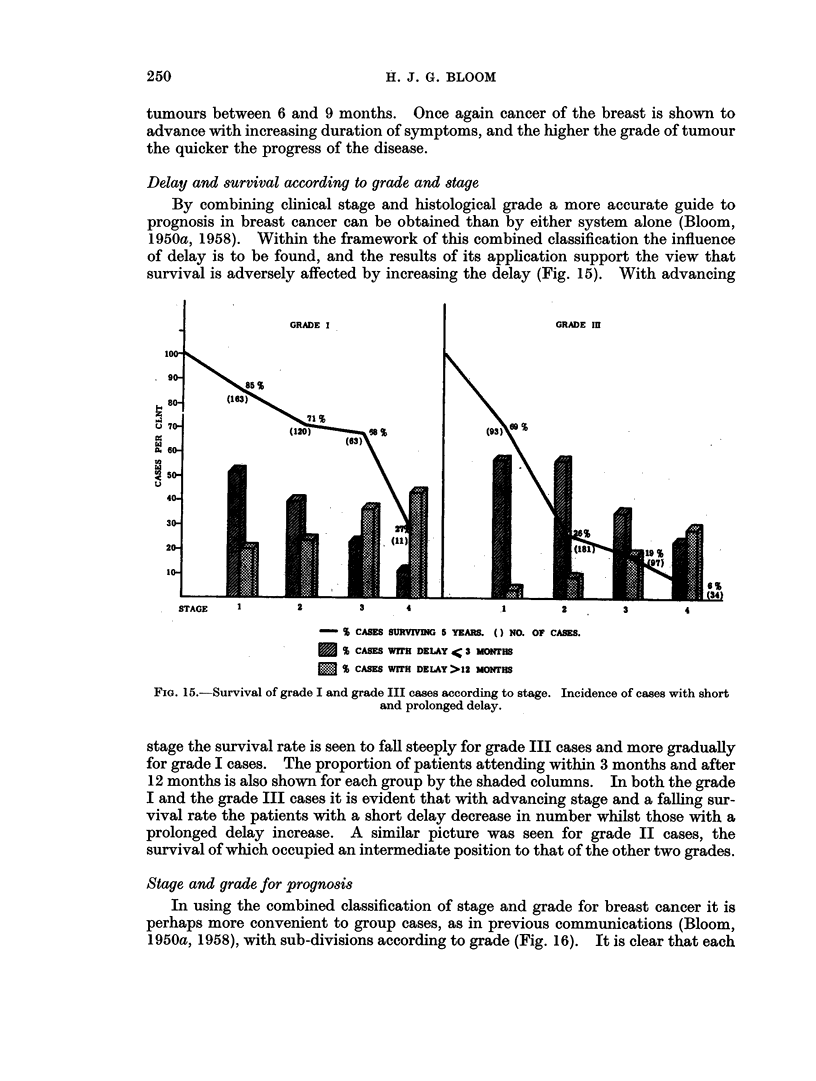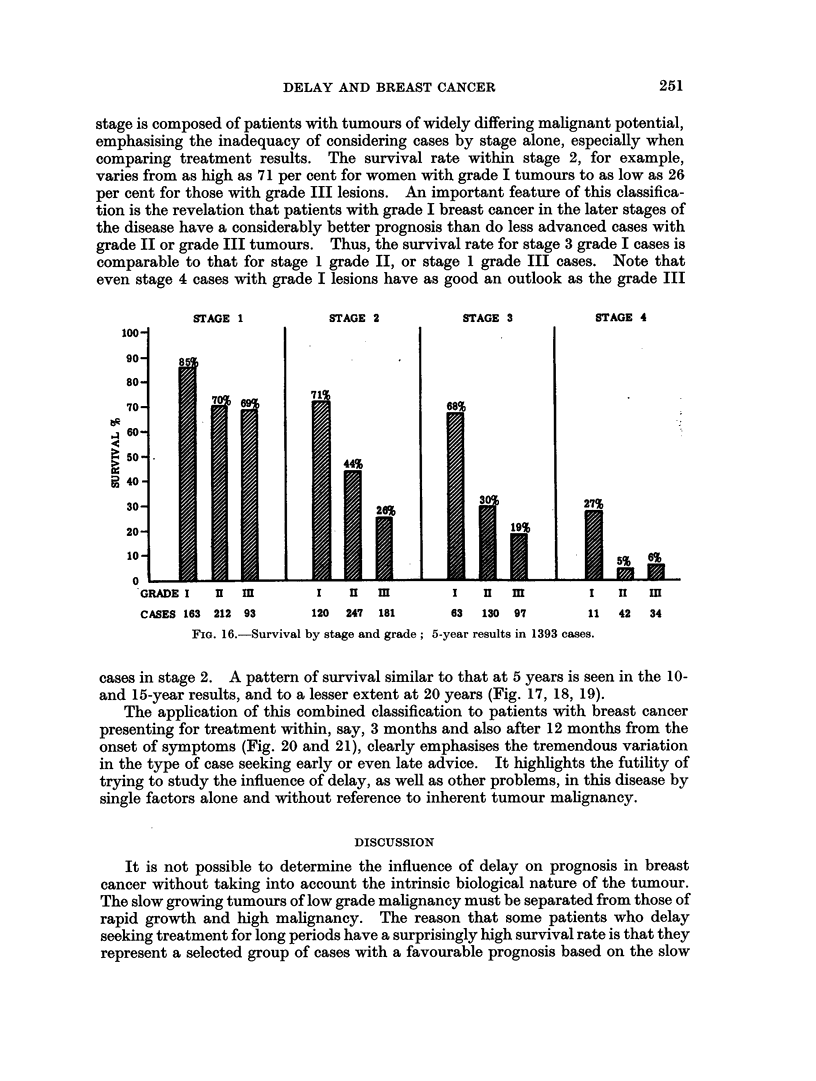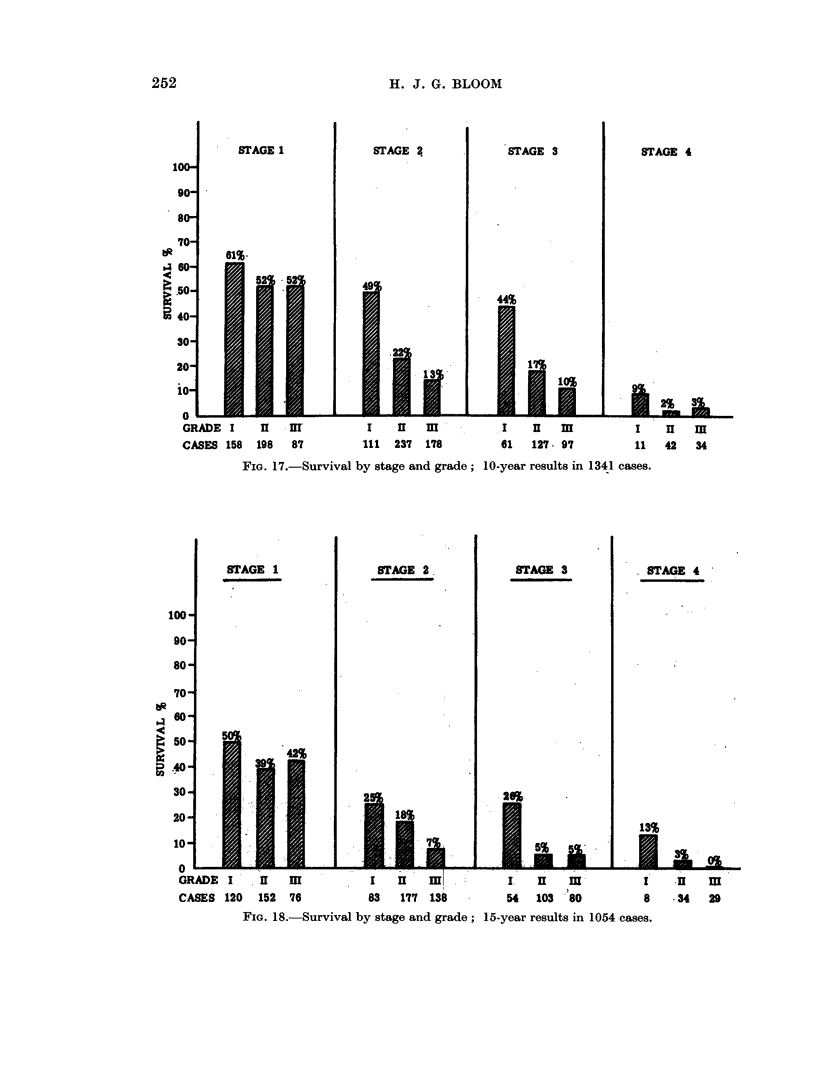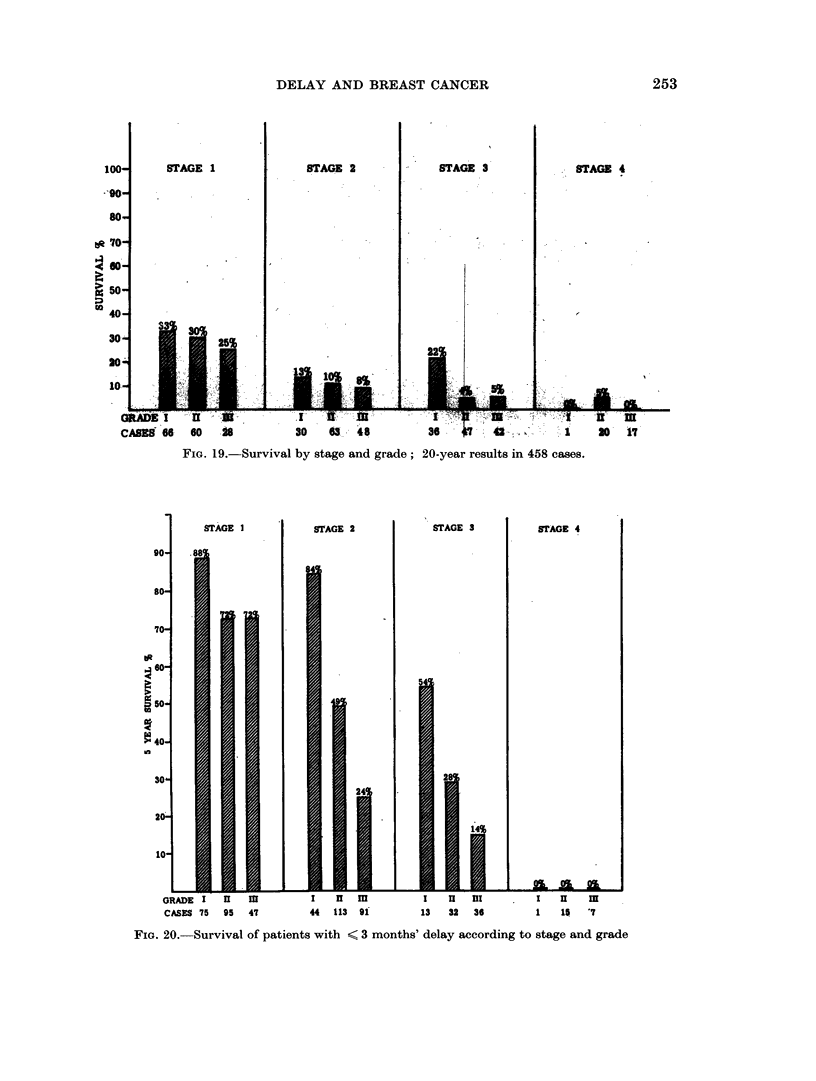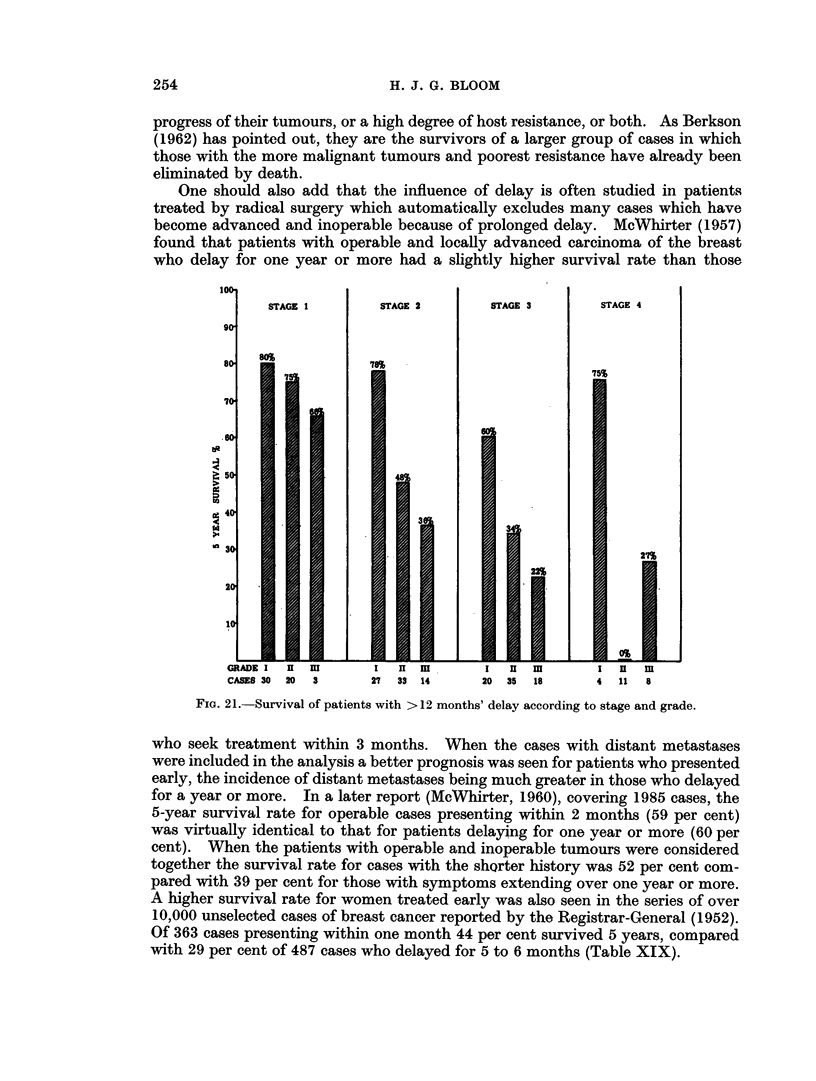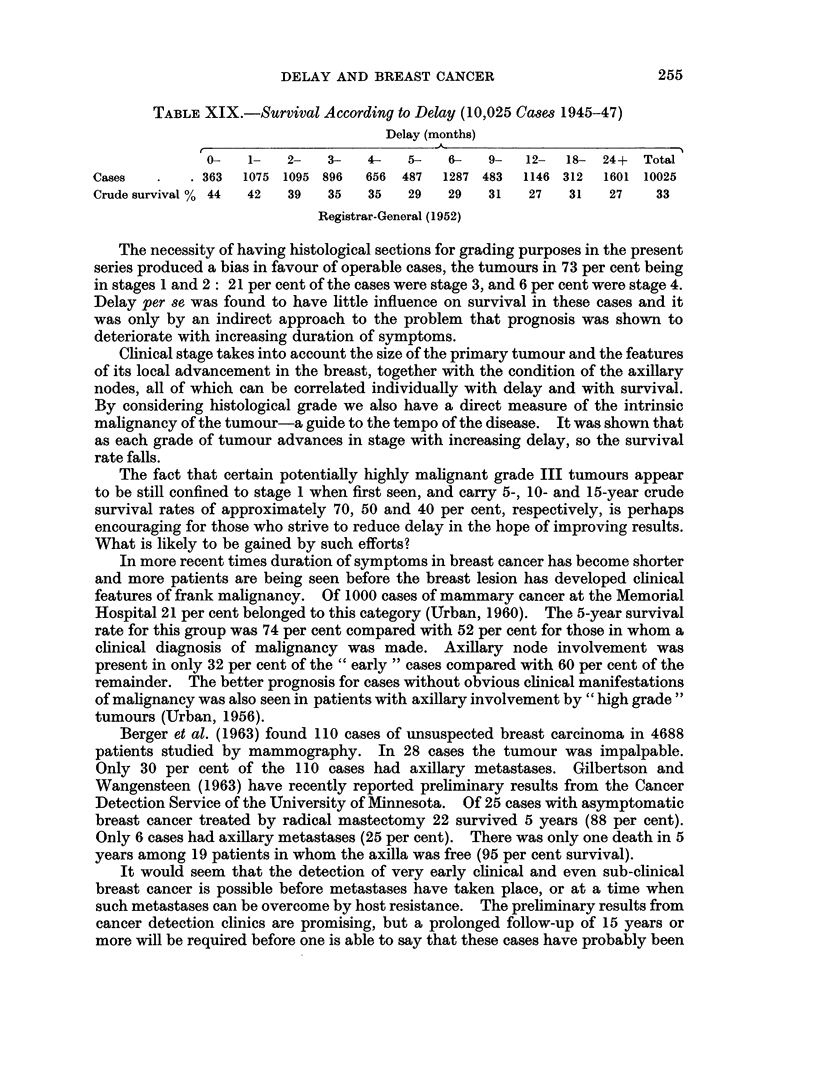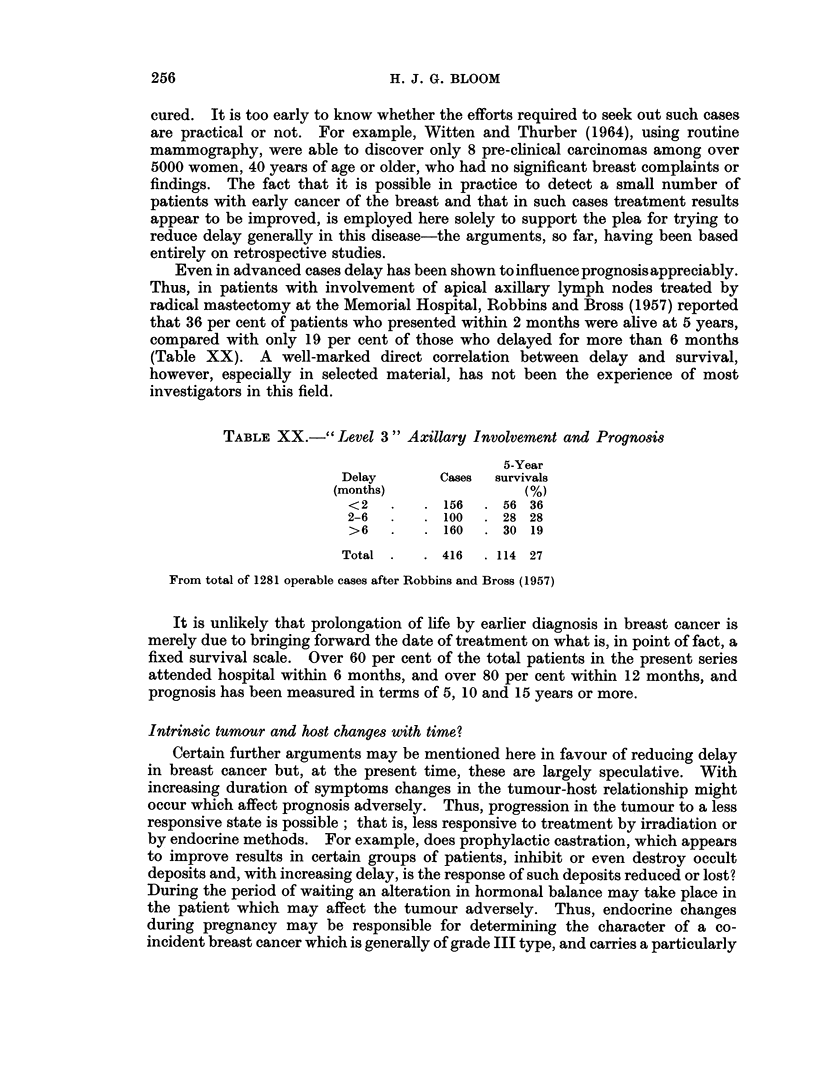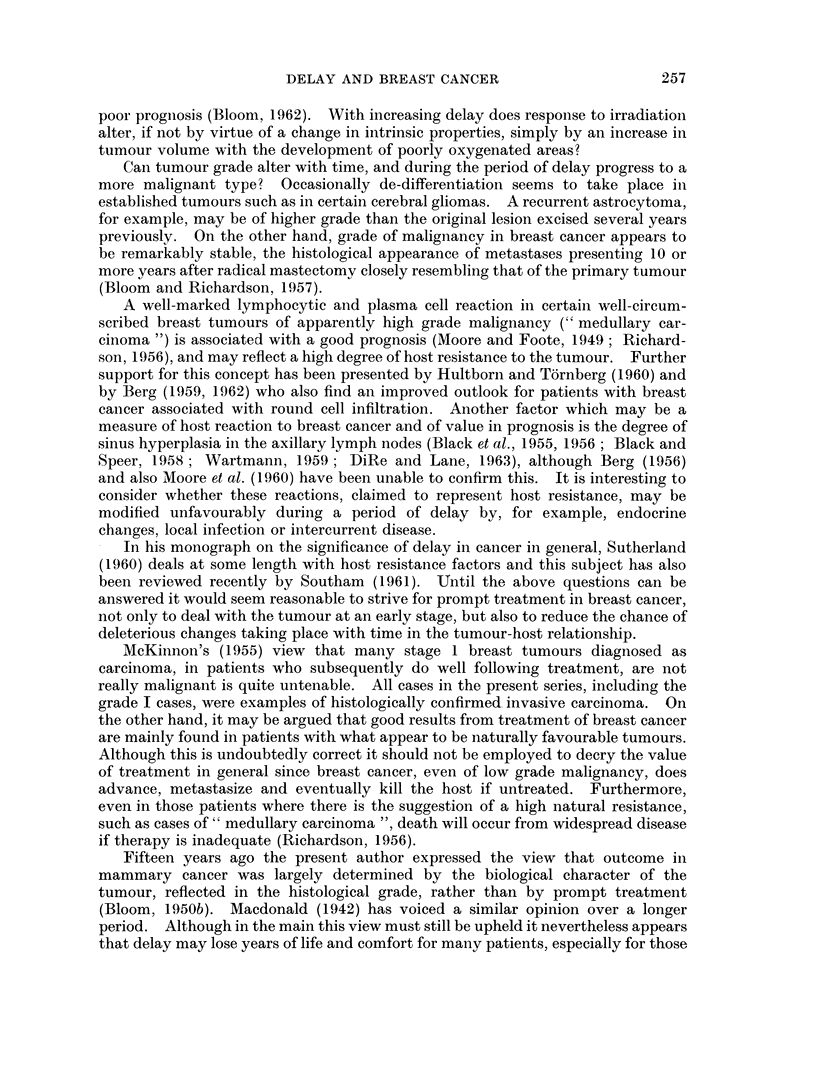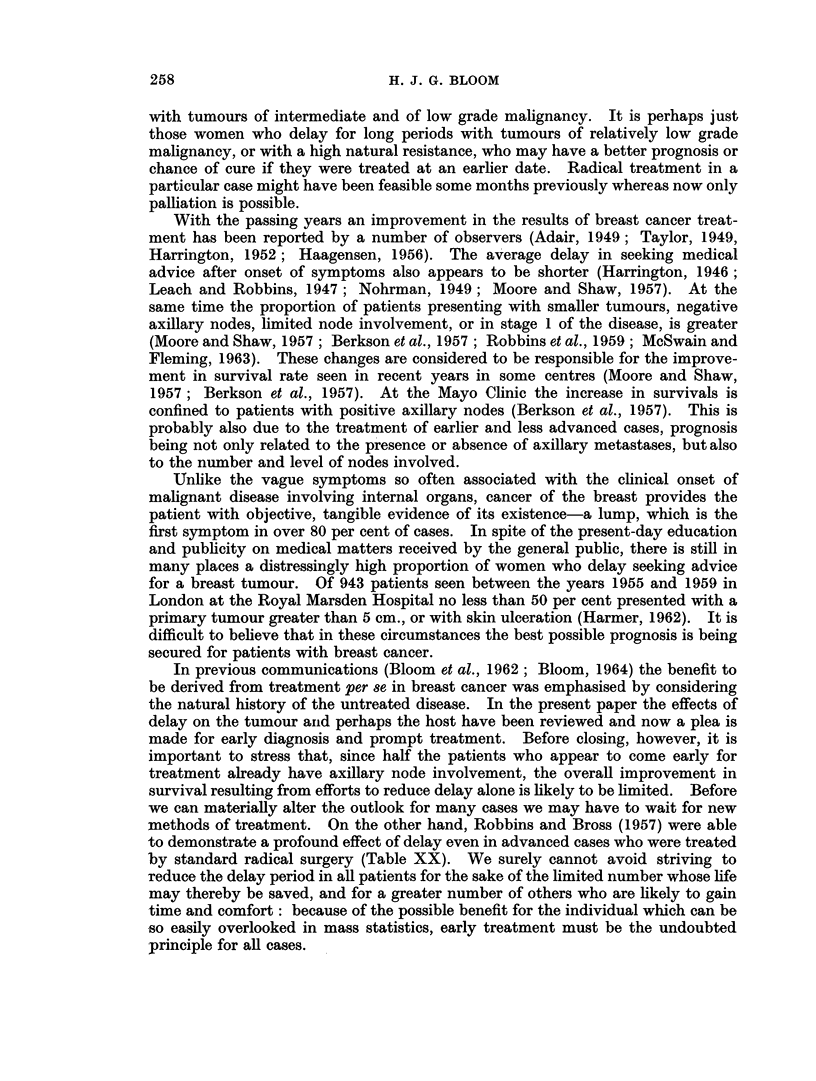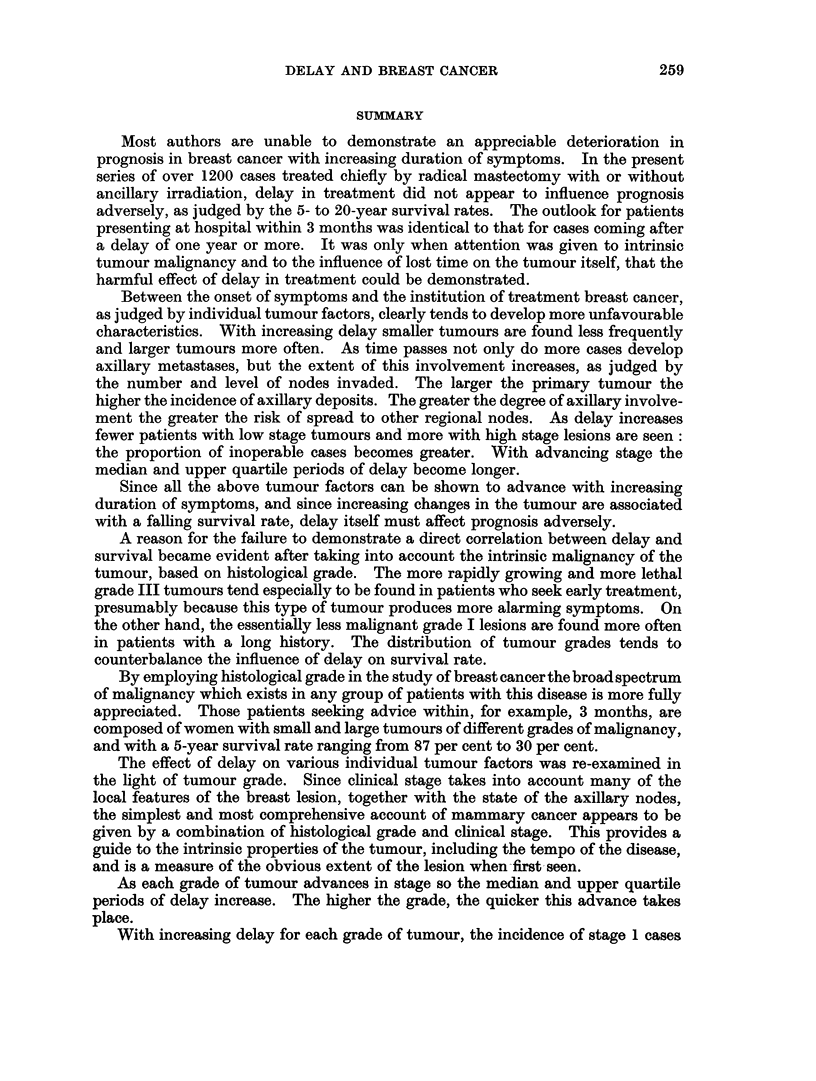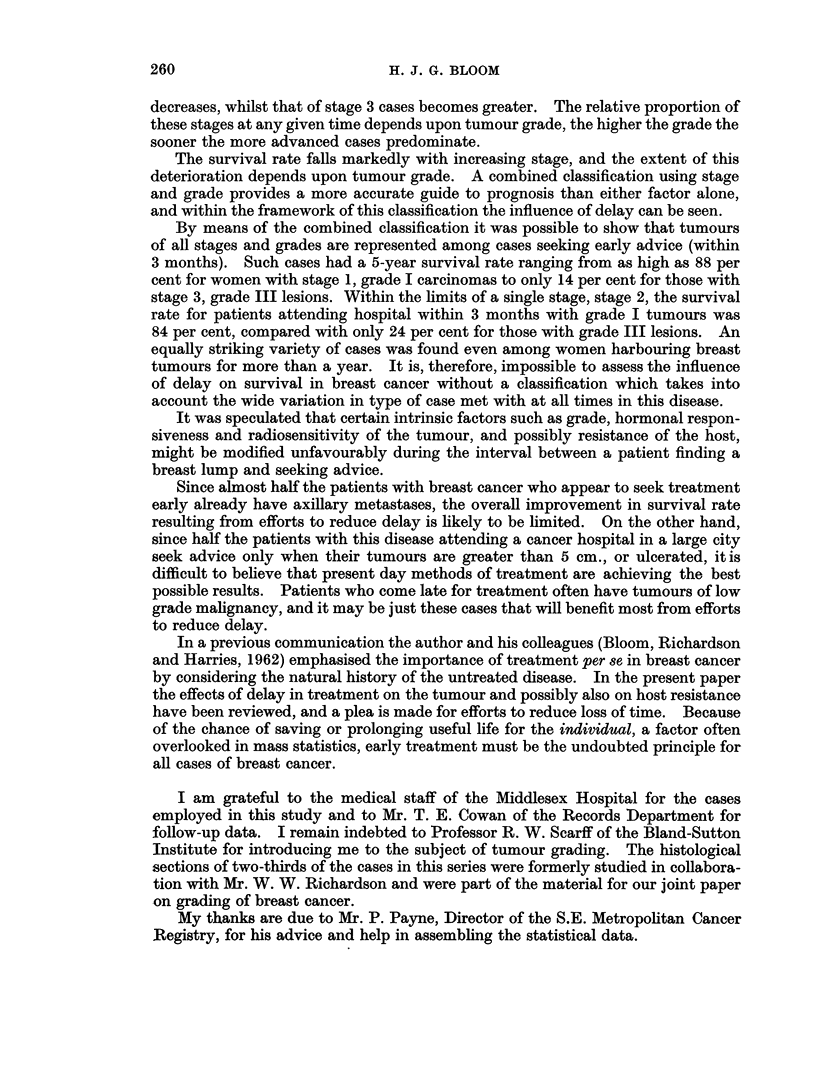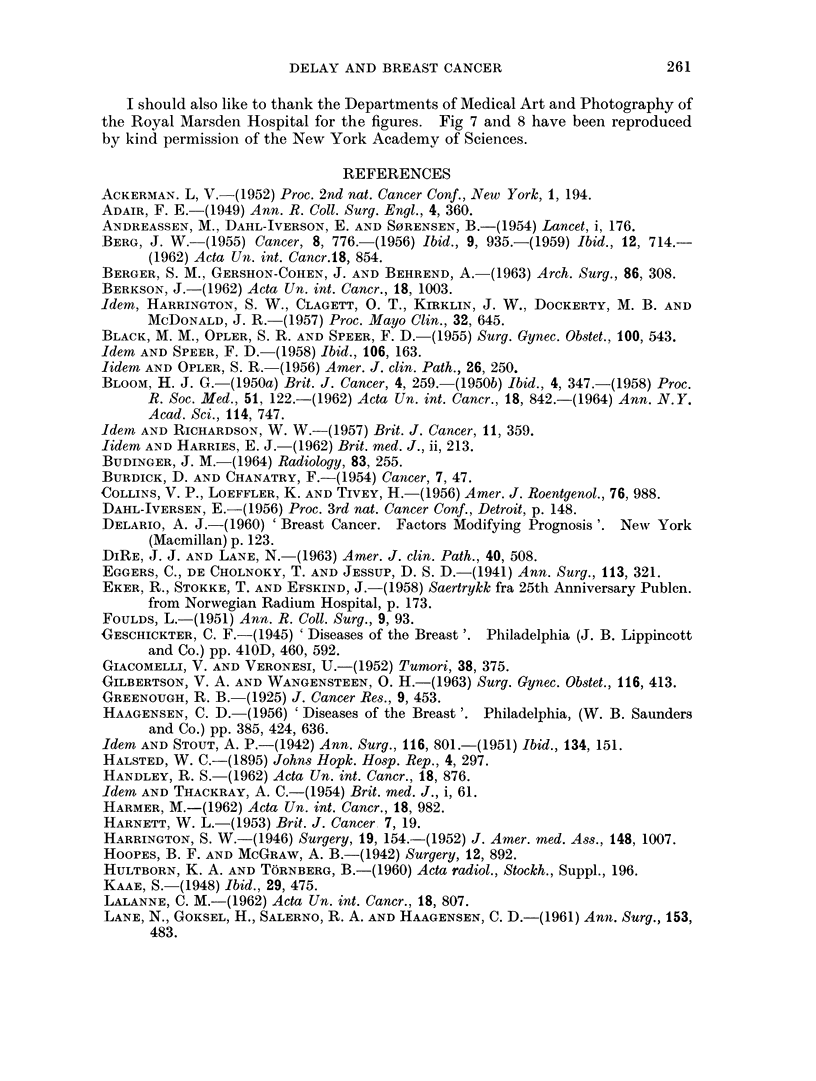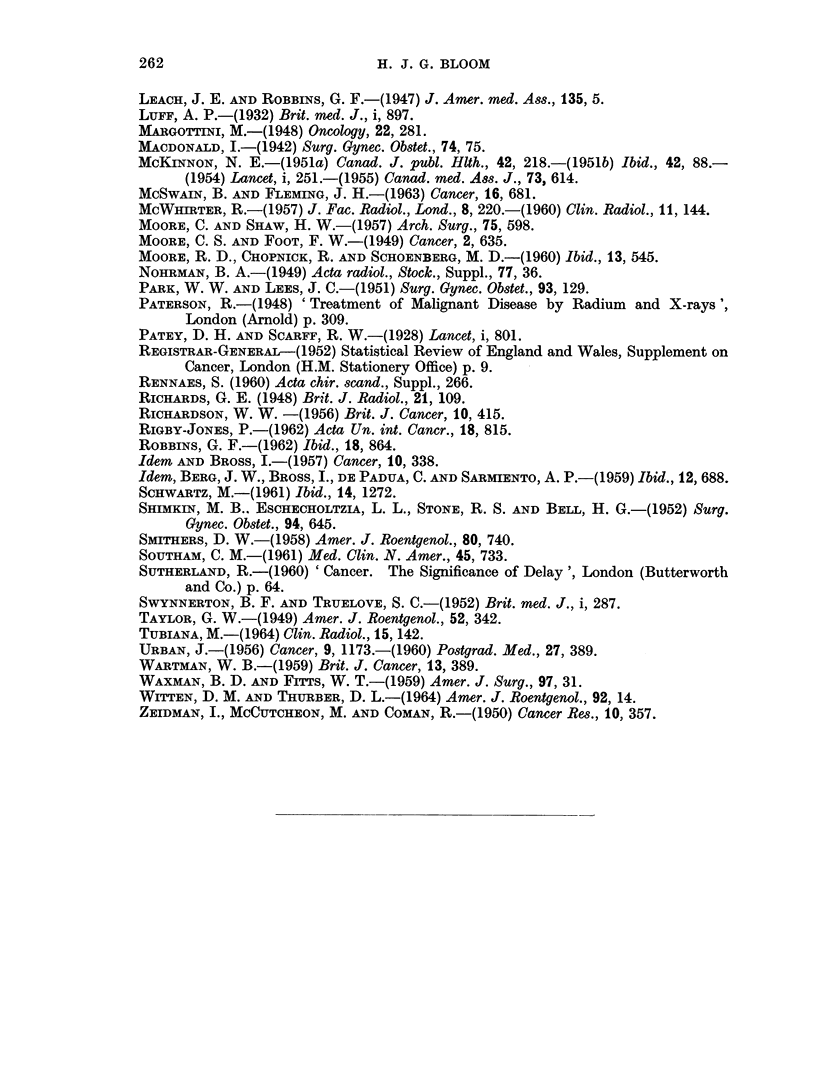# The Influence of Delay on the Natural History and Prognosis of Breast Cancer

**DOI:** 10.1038/bjc.1965.28

**Published:** 1965-06

**Authors:** H. J. G. Bloom


### THE INFLUENCE OF DELAY ON THE NATURAL HISTORY AND PROGNOSIS OF BREAST CANCER

### A STUDY OF CASES FOLLOWED FOR FIVE TO TWENTY YEARS*

H. J. G. BLOOM

*From the Royal Marsden Hospital and Institute of Cancer Research, Royal Cancer Hospital, London, S.W.3*

Received for publication January 13, 1965

WHENEVER the management of breast cancer is under discussion, as with cancer in general, it is only natural to stress the importance of early diagnosis and prompt treatment. Halsted (1895) considered that not even a single day should be lost in instituting treatment for breast cancer, and since then the general teaching has always been that the greater the delay the worse the prognosis. This view is often linked with the opinion that if patients receive early treatment the outlook is necessarily good. Efforts are, therefore, being made at the present time to persuade patients with symptoms suggestive of cancer to overcome their fears and to come forward promptly for medical advice. Some physicians, through cancer detection centres and by teaching self-examination of the breasts, are striving to find tumours before symptoms develop spontaneously.

A few years ago our complacency in demanding early treatment for cancer and our faith in its effectiveness was shaken by McKinnon (1951*a*, 1951*b*, 1954, 1955) in Canada, and by Park and Lees (1951) in England. The fact that survival rates for patients with breast cancer do not necessarily fall with increasing duration of symptoms has been taken as indicating that delay is of little significance in prognosis, and of further evidence that outcome is unaffected by treatment.

Conflicting reports are to be found in the literature regarding the influence of delay on prognosis in breast cancer. Whilst a few papers show a general reduction in survival rate with increasing duration of symptoms (Luff, 1932 ; Nohrman, 1949 ; Waxman and Fitts, 1959), and this is evident in a series of over 10,000 cases reported by the Registrar-General (1952) from England and Wales, most authors find only a slight or inconstant relationship between these factors. It is not uncommon to see a somewhat better prognosis for patients attending within a month or two compared with later cases, but in the experience of Delario (1960) and also Hultborn and Törnberg (1960) women treated within two weeks and within four weeks respectively appear to fare no better than those coming later. Haagensen and Stout (1942) found a reduction in the "cure" rate with increasing delay for their cases seen between 1915 and 1934, but were unable to confirm this observation in a later series between 1935 and 1942 (Haagensen and Stout, 1951). Many authors find a better prognosis for patients with a history extending over one to three years or more, compared with women having a shorter delay (Eggers *et al.*, 1941 ; Macdonald, 1942 ; Haagensen and Stout, 1942 ; Burdick and

* Based on an invited paper at the 10th Annual Clinical Conference of the Ontario Cancer Treatment and Research Foundation, Toronto, 8th November, 1963.

Chanatry, 1954 ; Haagensen, 1956 ; Berkson, 1962). In a previous report concerning 470 cases (Bloom, 1950*b*) the 5-year survival rate for patients attending within 3 months was 51 per cent, between 3 to 6 months 47 per cent, and after 12 months or more 52 per cent.

It is indeed a remarkable finding that patients who neglect the cancer in their breast for a year, or even longer, not infrequently appear to have a comparable or even better survival rate than do those women who seek advice after only a brief delay. This experience is not confined to breast cancer, and is also seen in reports concerning cancer of the stomach (Swynnerton and Truelove, 1952) and rectum (Harnett, 1953).

With so many conflicting results it was decided to investigate the effect of delay on the disease itself as well as on prognosis in a series of cases of breast cancer in which the influence of intrinsic tumour malignancy (histological grading) had already been studied (Bloom and Richardson, 1957), and in which a prolonged period of follow-up was now available.

### *Material*

The series for the present study consisted of 1411 cases seen between 1936 and 1949 at the Middlesex Hospital, London. These cases have been the subject of a previous inquiry in which the value of histological grading was investigated (Bloom and Richardson, 1957) and the importance of combining both stage and grade for prognosis emphasised (Bloom, 1958). Two patients, previously untraced, have been included in the present study. The stage was known in 1393 cases ; 468 were in stage 1, 548 in stage 2, 290 in stage 3, and 87 in stage 4 (Manchester system, Paterson, 1948). Eighty-four per cent of the patients were treated by radical or modified radical mastectomy with or without ancillary radiotherapy. The remainder were treated mainly by irradiation, surgery being limited to simple mastectomy, local excision or biopsy. All survival rates concerning these cases are crude figures, no correction being made for natural deaths.

Information regarding duration of symptoms was available in 1263 cases followed for 5 years, 1225 for 10 years, 951 for 15 years and 399 for 20 years. Of the total cases, 64 per cent attended hospital within 6 months and 82 per cent within one year. Less than half the patients (45 per cent) came within 3 months and only 16 per cent within one month ; 18 per cent delayed for more than one year and 8 per cent for more than 2 years.

The correlation between delay and survival is shown in Table I and Fig. 1. The 5-, 10- and 15-year survival rates are somewhat higher for patients treated within 3 months, but the gain does not exceed 10 per cent when compared with women who wait 3 to 6 months. After this time, the survival rate tends to increase slightly and, in patients delaying for more than 3 years, generally exceeds that for cases treated within 3 to 6 months. Since the case groups with a prolonged delay are small in number, especially in the series followed for 20 years, those patients with a delay exceeding one year have been grouped together in the lower part of the table to show a more consistent trend in prognosis. On the whole, the appearance of the survival curves in Fig. 1 is striking, only because of the very little change that occurs with increasing delay.

This is the type of evidence that is sometimes put forward to decry the value of cancer educational programmes, to proclaim the futility of cancer detection clinics, and to support the view that treatment of breast cancer does not materially

TABLE I.—*Delay and Prognosis*

| Delay | 5 Year Cases | 5 Year Survival (%) | 10 Year Cases | 10 Year Survival (%) | 15 Year Cases | 15 Year Survival (%) | 20 Year Cases | 20 Year Survival (%) |
|---|---|---|---|---|---|---|---|---|
| ⩽ 3 months | 571 | 53 | 556 | 36 | 425 | 24 | 180 | 16 |
| > 3–6 | 235 | 44 | 231 | 29 | 191 | 19 | 83 | 18 |
| > 6–12 ,, | 232 | 45 | 223 | 31 | 172 | 25 | 79 | 19 |
| >12–18 ,, | 58 | 52 | 55 | 29 | 40 | 28 | 19 | 16 |
| >18–24 ,, | 65 | 51 | 61 | 31 | 47 | 21 | 20 | 25 |
| > 2–3 years | 39 | 49 | 38 | 37 | 34 | 32 | 9 | 11 |
| > 3 ,, | 63 | 56 | 61 | 38 | 42 | 21 | 9 | 22 |
| Total | 1263 | 50 | 1225 | 33 | 951 | 24 | 399 | 18 |
| ⩽ 3 months | 571 | 53 | 556 | 36 | 425 | 24 | 180 | 16 |
| > 3–6 ,, | 235 | 44 | 231 | 29 | 191 | 19 | 83 | 18 |
| > 6–12 ,, | 232 | 45 | 223 | 31 | 172 | 25 | 79 | 19 |
| > 1 year | 225 | 52 | 215 | 33 | 163 | 25 | 57 | 19 |

influence survival. Does the information provided by simply correlating duration of symptoms and survival give the true picture concerning the effect of delay on prognosis? The purpose of this paper is to show that it does not.

The duration of symptoms in many cancer patients probably represents only a small fraction of the total pre-treatment duration of the primary tumour (Collins *et al.*, 1956; Schwartz, 1961). The obvious question to be answered then is whether carcinoma of the breast advances appreciably during the delay period and, if so, whether this degree of advancement is important in the ultimate outcome.

FIG. 1.—Delay and prognosis; total cases.

First, let us consider, step by step, the influence of delay on prognosis in the light of certain individual tumour factors, such as size of primary growth, lymph node involvement, the degree of this involvement, clinical stage and operability. Following this, attention will be drawn to the intrinsic malignancy of breast cancer, and emphasis will be placed on the value of histological grading with which it is hoped to try and explain some of the difficulties associated with breast cancer studies in general and with determining the true significance of delay in this disease.

### *Tumour Size*

#### *Size and delay*

Generally speaking, the trend is for smaller breast cancers to be found in patients who seek advice early, and for a progressively greater proportion of larger tumours to be associated with increasing delay (Macdonald, 1942 ; Kaae, 1948 ; Haagensen, 1956 ; Lalanne, 1962). This has been our experience at the Royal Marsden Hospital (Rigby-Jones, 1962), and in the cases reported here from the Middlesex Hospital (Table II). Since tumour size was expressed as inches in the majority of the notes this scale has been retained. Information regarding both tumour size and delay was available in 883 cases. Approximately half of the patients presenting within one month had tumours less than one inch diameter whilst in 11 per cent the size exceeded 2 inches ; in patients delaying more than 12 months 30 per cent had the small tumours and 24 per cent the larger ones.

TABLE II.—*Delay and Tumour Size*

| Delay (months) | Tumour size (inches) | | | | |
|---|---|---|---|---|---|
| | ≤½ in. (%) | >½≤1 in. (%) | >1≤2 in. (%) | >2 in. (%) | Total (%) |
| ≤1 | 10 | 39 | 40 | 11 | (161) 100 |
| ≤3 | 7 | 37 | 41 | 15 | (411) 100 |
| >3≤6 | 10 | 36 | 36 | 18 | (171) 100 |
| >6≤12 | 7 | 24 | 49 | 20 | (152) 100 |
| >12 | 7 | 23 | 46 | 24 | (149) 100 |

#### *Size and survival*

The 5- to 20-year results according to tumour size are presented in Fig. 2. Survival rate is seen to fall rapidly with increasing tumour diameter. Patients with tumours, for example, of half-an-inch or less have a 5- and 10-year survival rate of 70 and 46 per cent respectively, compared with only 13 per cent and 5 per cent for those with growths exceeding 3 inches. These findings are in keeping with those of other observers such as Eggers *et al.* (1941), Geschickter (1945), Adair (1949), Burdick and Chanatry (1954), McWhirter (1957, 1960), Eker *et al.* (1958) and Hultborn and Törnberg (1960). Lane and his colleagues (1961), who have recently drawn attention to the prognostic significance of the gross contour of the primary tumour, found that size influenced survival only in those tumours with an irregular outline and was without effect on cases with well-delineated lesions. Only one of 37 patients in the present series with a tumour greater than 3 inches in diameter was alive 15 years after treatment, whereas 37 per cent of 65 women with lesions of half-an-inch or less survived for this time (Fig. 2).

FIG. 2.—Size and prognosis.

*Size, delay and survival*

By considering size of the breast tumour and duration of symptoms it is possible to obtain some idea of tumour growth rate. Richards (1948) studied this aspect of breast cancer and obtained a good correlation with prognosis, the faster the growth rate the lower the survival rate. Both tumour size and delay have been correlated with prognosis in the present series (Fig. 3). The effect of the faster growing tumours is seen in the survival rate for cases presenting within 3 months which, as one might expect, shows the greatest reduction with increasing tumour size. The more slowly growing lesions produce the least change in survival rate, and this is seen in cases presenting after a delay of 12 months or more.

It is evident that the outlook for women who seek treatment early varies considerably, depending upon the size of their tumour at the first examination (Fig. 3). Thus, of patients attending within 3 months with tumours of half-an-inch or less, 86 per cent are alive at 5 years, compared with only 21 per cent of those with lesions greater than 2 inches. After a delay of 12 months, 73 per cent of patients with small carcinomas are alive, compared with 37 per cent of those harbouring large tumours.

These findings point to the importance of considering " intrinsic tumour malignancy " when trying to evaluate the influence of delay in breast cancer, a matter to be considered in detail in later sections of this communication.

### *Lymph Node Involvement*

*Lymph node involvement and delay*

Although the risk of axillary node involvement is greater with increasing duration of symptoms, this often amounts to no more than a trend (Haagensen and

Fig. 3.—Delay, size and prognosis.

Stout, 1942, 1951 ; Hoopes and McGraw, 1942 ; Park and Lees, 1951 ; Burdick and Chanatry, 1954). In the present series the histological state of the axillary nodes and the duration of symptoms were known in 1047 cases. Although the relationship is not consistent, the general direction is towards a slightly higher

TABLE III.—*Delay and Axillary Involvement*

| Delay | Cases | Axilla involved* % | |
|---|---|---|---|
| ≤ 1 month | 196 | 59 | |
| >1≤ 2 ,, | 162 | 61 | 58% |
| >2≤ 3 ,, | 133 | 52 | |
| >3≤ 4 ,, | 69 | 61 | |
| >4≤ 6 ,, | 134 | 66 | 64% |
| >6≤12 ,, | 181 | 63 | |
| >1≤ 2 years | 94 | 67 | 67% |
| >2≤ 3 ,, | 31 | 61 | |
| > 3 ,, | 47 | 68 | 68% |
| Total | 1047 | 61 | |

* Histological assessment.

incidence of axillary metastases with increasing delay (Table III), The actual extent or degree of axillary node involvement can also be related to delay as shown by the incidence of apical (" level 3 ") axillary metastases in the report by Robbins and Bross (1957) from the Memorial Hospital (Table IV).

TABLE IV.—*Degree of Axilla Involvement According to Delay*

| Delay (months) | Cases | " Level 3 " axilla involvement |
|---|---|---|
| <2 | 550 | 28% |
| 2–6 | 336 | 30% |
| >6 | 395 | 40% |

Robbins and Bross (1957)

Involvement of the internal mammary chain has been correlated with the extent of axillary node metastases by Handley and Thackray (1954) at the Middlesex Hospital (Table V), and also by Tubiana (1964) at the Gustave Roussy Institute (Table VI). Finally, it is in the presence of axillary metastases that the stage is set for spread to the supraclavicular nodes.

TABLE V.—*Internal Mammary Node Metastases According to Degree of Axilla Involvement*

| Degree of axilla involvement | Cases | With internal mammary metastases (%) |
|---|---|---|
| Nil | 57 | 14 |
| Slight | 23 | 26 |
| Moderate | 32 | 37 |
| Heavy | 34 | 65 |

Handley and Thackray (1954)

TABLE VI.—*Internal Mammary Node Involvement*

| Axillary nodes +ve | 0 | 1 | 2 | 3 | 4–5 | 6–7 | 8 |
|---|---|---|---|---|---|---|---|
| Number of cases | 35 | 29 | 23 | 17 | 18 | 19 | 23 |
| Internal mammary nodes | (%) | (%) | (%) | (%) | (%) | (%) | (%) |
| —ve | 91 | 76 | 65 | 71 | 50 | 58 | 52 |
| +ve | 9 | 24 | 35 | 29 | 50 | 42 | 48 |

Tubiana (1964)

*Lymph node involvement and primary tumour size*

A correlation exists between primary tumour size and axillary node metastases, the larger the primary lesion the greater the likelihood of such metastases being present (Eggers *et al.*, 1941; Ackerman, 1952; Berg, 1955; Rennaes, 1960; Lane *et al.*, 1961). This relationship is more striking than that between delay and tumour size, and also between delay and axillary involvement. In the present series information regarding both tumour size and histological assessment of the axillary nodes was available in 853 cases. Practically 50 per cent of the patients with tumours half-an-inch or less in diameter had axillary metastases, compared with 94 per cent of those with tumours exceeding 3 inches (Table VII).

Giacomelli and Veronesi (1952) and also Haagensen (Budinger, 1964) have found that, with increasing size of breast tumour, metastases are more frequent in the internal mammary lymph nodes.

TABLE VII.—*Primary Tumour Size and Axillary Involvement*

| Size (inches) | Cases | Axillary involvement* (%) |
|---|---|---|
| $\leqslant\frac{1}{2}$ | 70 | 49 |
| $>\frac{1}{2}\leqslant 1$ | 280 | 49 |
| $>1\leqslant 2$ | 363 | 61 |
| $>2\leqslant 3$ | 104 | 76 |
| $>3$ | 36 | 94 |
| Total | 853 | 59 |

* Histological assessment.

Thus, with increasing primary tumour size, which is related to delay, or with increasing delay *per se*, there is not only a higher incidence of axillary node metastases, but the degree of this involvement is also greater. With increasing delay and progressive axillary involvement, internal mammary and supraclavicular node metastases become more frequent.

*Lymph node involvement and survival*

There is general agreement that in breast cancer the outlook for women with axillary metastases is considerably worse than for patients without this complication. In the present series information regarding the condition of the axillary nodes was available in 1311 cases, in 89 per cent of which the assessment was from histological sections (Table VIII). The 5-year survival rate for women without axillary metastases was twice as great as for cases with such metastases ; the corresponding prognosis at 10, 15 and 20 years, when the axilla was free, was better by a factor of three. four and nearly five. respectively.

TABLE VIII.—*Axilla Involvement and Prognosis*

| | Results | | | | | | | |
|---|---|---|---|---|---|---|---|---|
| | 5 years | | 10 years | | 15 years | | 20 years | |
| Axilla | Cases | Survival (%) | Cases | Survival (%) | Cases | Survival (%) | Cases | Survival (%) |
| 0 | 520 | 73 | 497 | 55 | 384 | 43 | 172 | 31 |
| + | 791 | 36 | 763 | 19 | 595 | 11 | 217 | 7 |
| Total | 1311 | 50 | 1260 | 33 | 979 | 24 | 389 | 18 |

Less attention has been given to the fact that the actual number of axillary glands involved, and the extent of this involvement, markedly affects prognosis (Adair, 1949 ; Haagensen, 1956 ; Hultborn and Törnberg, 1960 ; Robbins, 1962), (Tables IX and X). To determine the condition of the axilla accurately requires an adequate dissection of this region during radical mastectomy, a careful search for lymph nodes in the post-operative specimen, and subsequent examination of multiple histological sections of these nodes. In many hospitals only a limited examination of the axillary contents is carried out but, even so, a useful guide to prognosis may be obtained from such material. A study was made of the routine histological lymph node sections in the first 400 cases of the Middlesex series, which included patients treated at Sector and other hospitals during the War years. Although only a very rough assessment of the degree of axillary involve-

TABLE IX.—*Degree of Axilla Involvement and Prognosis*

| Axillary nodes +ve | Cases | 5-year survival (%) | 5-year local recurrence (%) |
|---|---|---|---|
| 1–2 | 67 | 75 | 8 |
| 3–7 | 62 | 53 | 21 |
| 8+ | 61 | 26 | 48 |
| Total | 190 | 52 | 25 |

Haagensen (1956)

TABLE X.—*Degree of Axilla Involvement and Prognosis*

| Axilla involvement | Cases | Survival rate 5 years (%) | 10 years (%) | 15 years (%) |
|---|---|---|---|---|
| O | 574 | 80 | 58 | 44 |
| Level 1 +ve | 164 | 61 | 41 | 30 |
| Level 2 +ve | 159 | 45 | 30 | 23 |
| Level 3 +ve | 363 | 28 | 15 | 10 |

Robbins (1962)

ment was possible in most cases from the limited histological sections available, a correlation with survival was obtained (Table XI).

TABLE XI.—*Axilla Involvement and Prognosis*

| Axilla involvement (histological) | Cases | 5-year survivors | (%) |
|---|---|---|---|
| O | 175 | 125 | 66 |
| ++ | 86 | 38 | 44 |
| +++ | 138 | 35 | 25 |
| Total | 399 | 198 | 50 |

O = Nodes free
++ = Moderate involvement of 1–2 nodes
+++ = More extensive involvement

Metastases in the internal mammary lymph nodes alone is uncommon, and appears to effect prognosis in much the same way as does axillary involvement. On the other hand, when deposits are present in both these regions the outlook seems to be exceedingly poor ; only 1 of 29 such cases in Handley's (1962) series survived 10 years.

The discovery of occult supraclavicular metastases in 10 to 20 per cent of patients with axillary metastases, otherwise suitable for radical surgery (Dahl-Iverson, 1956 ; Margottini, 1948 ; Haagensen, 1956), is a grave sign : only 3 of 17 such cases reported by Andreassen *et al.* (1954) survived 5 years.

At the beginning of this paper it was not possible to show that delay influenced survival appreciably. We now find that the factor of delay can be related to the presence and to the extent of regional lymph node involvement, and that these in turn are closely related to prognosis.

### *Extent of the Disease*

### *Stage and delay*

Clinical staging is a convenient way of combining several local factors concerning the primary tumour together with the state of the axillary lymph nodes. It is among those patients with a prolonged delay that a greater proportion of tumours in the more advanced stages is to be found. A good correlation between stage and delay is seen in the cases studied by Nohrman (1949), Shimkin *et al.* (1952) (Table XII) and Lalanne (1962).

TABLE XII.—*Delay According to Stage*

| Stage (Portmann) | Cases | Median period of delay (months) |
|---|---|---|
| 1 | 333 | 4·0 |
| 2 | 296 | 5·5 |
| 3 | 310 | 11·5 |
| 4 | 99 | 25·0 |

Shimkin *et al.* (1952)

In the present material information regarding both stage (Manchester system) and delay was available in 1255 cases. A correlation between stage and delay is seen when we consider the time in which a given proportion of patients attend for treatment (Table XIII). For example, the upper quartile (when 75 per cent of patients attend) for stage 1 cases is 7·3 months ; stage 2 cases, 7·5 months ; stage 3 cases, 13 months and stage 4 cases, 19 months.

TABLE XIII.—*Delay According to Stage*

| Group | Delay (months) | | | |
|---|---|---|---|---|
| | Stage 1 419 cases | Stage 2 495 cases | Stage 3 267 cases | Stage 4 74 cases |
| Lower quartile . . | 1·2 | 1·2 | 2·4 | 2·5 |
| Median . . . | 2·9 | 3·0 | 6·1 | 5·8 |
| Upper quartile . . | 7·3 | 7·5 | 13·0 | 19·0 |

Lower quartile : time (months) within which 25% of cases attended
Median : ,, ,, ,, ,, 50% ,, ,, ,,
Upper quartile : ,, ,, ,, ,, 75% ,, ,, ,,

With increasing duration of symptoms there is some variation in the incidence of the different stages (Fig. 4), but up to 3 years the proportion of patients with tumours in stage 1 or stage 2 clearly diminishes, whilst cases with lesions in the more advanced stages increase. Thus of the 569 cases attending within three months 38 per cent are confined to stage 1, whilst 18 per cent have extended to stages 3 or 4 (Table XIV). The corresponding figures for 100 cases with a delay

TABLE XIV.—*Incidence of Cases by Stage According to Delay*

| Delay | Cases | Stage 1* (%) | Stage 2* (%) | Stage 3+4* (%) |
|---|---|---|---|---|
| ⩽ 3 months . . . | 569 . | 38 . | 44 . | 18 |
| 6–12 ,, . . | 228 . | 32 . | 34 . | 34 |
| > 2 years . . . | 100 . | 21 . | 30 . | 49 |

* Manchester staging exceeding 2 years is 21 per cent and 49 per cent respectively. For an intermediate period of delay of between 6 and 12 months the proportion of early and advanced cases is practically equal. A similar correlation between stage and duration of symptoms has also been shown in cases at the Royal Marsden Hospital (Smithers, 1958).

Among the 62 patients in the present series with a delay exceeding 3 years there is a sharp increase in the proportion of stage 2 tumours (Fig. 4). This is due, presumably, to a preponderance of carcinomas of relatively slow growth rate and of low biological potential since these cases, in spite of neglect, have already been

FIG. 4.—Stage incidence according to delay.

able to survive a considerable length of time without treatment. Support for this explanation is to be found in a later section of this paper when tumour grade is considered in relation to delay (Table 17).

These results agree in the main with those reported by the Registrar-General (1952) in an unselected series of over 21,000 cases of breast cancer registered in England and Wales between 1945 and 1949. The median delay for patients with tumours confined to the breast was 4·1 months, locally advanced lesions 9·1 months, and for those with distant metastases 11·4 months. The well-defined correlation between stage of disease and duration of symptoms in the Registrar-General's (1952) report has been confirmed in a second large series of cases from the same source, registered between 1950 and 1954 (Spicer and Lipworth, 1965, personal communication). Lalanne (1962) also found a greater incidence of distant metastases with increasing duration of symptoms : for a delay of less than 2 months, 6 per cent of his patients had metastases compared with 12 per cent of those waiting 6 to 12 months, and 26 per cent for a delay exceeding one year. In this respect the experimental work of Zeidman and his colleagues (1950) is of interest. These workers have shown in animals that the longer a tumour is present the greater the number of emboli released, as judged by the number of metastases appearing in the lungs.

FIG. 5.—Stage and survival.

It is evident that mammary cancer tends to progress to a more advanced stage during the interval between a woman first becoming aware of a breast lump and the time she finally comes under medical care.

*Stage and survival*

There is general agreement that survival in breast cancer falls profoundly with increasing clinical stage of the disease. In the present series information regarding stage was available in 1393 cases. The deterioration in prognosis according to stage is seen in the 5-, 10-, 15- and 20-year results (Fig. 5).

### *Inoperability and Delay*

Closely linked with clinical stage is the question of operability. With increasing delay fewer patients are suitable for radical mastectomy. Kaae (1948) found that of 84 cases seen within 2 weeks of onset of symptoms all were operable, compared with 77 per cent of 140 patients presenting within 3 to 12 months. More recently, Rennaes (1960) reported that of 421 cases presenting within one month at the Norwegian Radium Institute, 91 per cent were operable compared with only 52 per cent of 275 cases who waited for more than 12 months. The effect of delay on radical treatment in a large series of unselected cases is to be found in the Registrar-General's (1952) report covering over 21,000 patients in England and Wales (Table XV).

TABLE XV.—*Cases Receiving Radical Treatment According to Delay*

| Delay (months) | $<2$ (%) | $<6$ (%) | $<12$ (%) | $>12$ (%) |
|---|---|---|---|---|
| Receiving radical treatment . | 88 | 77 | 65 | 61 |

Registrar-General (1952)
21,508 cases
(1945–1949)

As far as we have gone the conclusion must be that with increasing delay the primary tumour tends to increase in size, the axillary and internal mammary lymph nodes to become involved, the stage of the disease to advance, distant metastases to be a greater risk, and operability to diminish. It would, therefore, seem logical to expect the outlook for patients with breast cancer who come late for treatment to be distinctly unfavourable when delay itself is correlated with survival. We have seen that this is not the case, the survival often remaining remarkably uniform in spite of increasing delay (Fig. 1). What factors may account for this discrepancy?

So far, no attention has been given to the inherent properties of the tumour itself, nor to possible local and systemic resistance factors on the part of the host. It is true that such factors as size of tumour, growth rate, axillary involvement and stage of disease reflect tumour potential, but only to a limited extent. Elsewhere, (Bloom, 1950*a*, 1958) it has been shown that cases in any given stage of the disease are composed of patients with tumours of widely differing degrees of malignancy, the latter feature being conveniently expressed in terms of three histological grades.

The concept of biological potential is complex in that although more malignant tumours tend to increase in size, infiltrate quickly and disseminate early, these three features need not be in step (Foulds, 1951). Thus, a small breast tumour may already have given rise to metastases by the time it is found by the patient. On the other hand, a large tumour may still be confined to the breast, whilst other growths become locally advanced with involvement of the axillary lymph nodes but without distant metastases.

### *Histological Grading*

Many reports indicate that the potential malignancy of breast cancer is generally reflected in its histological architecture (Greenough, 1925 ; Patey and Scarff, 1928 ; Geschickter, 1945 ; Harrington, 1946, 1952 ; Bloom, 1950*a*, 1950*b* ; Black *et al.*, 1956 ; Haagensen, 1956 ; Bloom and Richardson, 1957).

There has been much argument as to the prognostic significance of various histological criteria, both parenchymal and stromal. The most consistent results have been obtained by considering the degree of tubular differentiation, regularity of nuclear size, shape and staining, and the frequency of hyperchromatic and mitotic figures. Taking the histological picture as a whole, each tumour can be classified as being of low (grade I), intermediate (grade II) or high (grade III) malignancy. These three groups merely represent arbitrary subdivisions of what is, in fact, a continuous scale of malignancy. This subject and its problems have been reviewed in detail elsewhere (Bloom, 1950*a* ; Bloom and Richardson, 1957).

FIG. 6.—Grade and prognosis.

Survival in the present series of cases according to grade of malignancy at 5-, 10-, 15- and 20-years from the time of treatment is shown in Table XVI and Fig. 6. A clear difference is seen for the three grades, the survival rate for patients with grade I tumours being between two and three times greater than that for grade III cases. Most of the deaths from grade III tumours take place in the first 5 years, whereas the mortality of grade I cases occurs at a considerably slower and more uniform rate over the entire 20 years (Fig. 6).

The importance of grading breast cancer is further emphasised by a recent study of *untreated* cases from the past records of the Middlesex Hospital Cancer Charity between 1805 and 1933 (Bloom *et al.*, 1962). In this series there were 86 cases, seen between 1902 and 1933, in which histological material was available. Although the numbers in each group are small a striking difference is seen in the

TABLE XVI.—*Grade and Prognosis*

| Grade | 5-year results (1936–49) | | | 10-year results (1936–49) | | | 15-year results (1936–47) | | | 20-year results (1936–42) | | |
|---|---|---|---|---|---|---|---|---|---|---|---|---|
| | Cases | Survivals | (%) | Cases | Survivals | (%) | Cases | Survivals | (%) | Cases | Survivals | (%) |
| I | 363 | 272 | 75 | 347 | 180 | 52 | 271 | 97 | 36 | 135 | 34 | 25 |
| II | 640 | 301 | 47 | 613 | 178 | 29 | 473 | 96 | 20 | 192 | 27 | 14 |
| III | 408 | 131 | 32 | 399 | 79 | 20 | 326 | 45 | 14 | 135 | 13 | 10 |
| Total | 1411 | 704 | 50 | 1359 | 437 | 32 | 1070 | 238 | 22 | 462 | 74 | 16 |

natural history of tumours of grade I and grade III malignancy (Fig. 7). At 5 years 22 per cent of grade I cases were alive, whereas all those with grade III tumours were dead. After 10 years, 9 per cent of grade I and 3 per cent of grade II cases were still alive. The mean survival of grade I cases was 47·3 months (range 6 to 166) ; grade II, 39·2 months (range 5 to 122) ; grade III, 22 months (range 2

FIG. 7.—Untreated breast cancer ; histological grade and survival, Middlesex Hospital, 1902–1933 (86 cases)

to 53). The mean duration of symptoms and of life after admission to hospital was more than twice as long for grade I as it was for grade III cases (Fig. 8) (Bloom, 1964).

The influence of axillary node involvement on overall survival has already been considered (Table VIII). The state of these nodes is now correlated with histological grade for prognosis (Fig. 9). For clarity the survival of cases with tumours of low (grade I) and of high (grade III) malignancy only are shown in this figure. Grade II cases occupied a well-defined intermediate position in the group with axillary node metastases, but showed a comparable survival curve to grade III cases when the axilla was free. The striking difference in outlook for patients with axillary metastases, depending on whether they have grade I or grade III tumours, is a clear example of the limited information which may be given in breast cancer when only one factor of the disease is studied and when inherent malignancy is neglected. Thus, the 10-year survival rate for all patients with axillary node involvement is 19 per cent (Table VIII), but within this group 42 per cent of grade

FIG. 8.—Untreated breast cancer.

FIG. 9.—Survival according to grade and axilla involvement.

I cases are alive compared with a mere 9 per cent of those with grade III lesions (Fig. 9). At 20 years, however, the difference in survival rate for these two grades in the presence of axillary metastases is much smaller. When the axilla is free, the difference between grade I and grade III cases is less marked, but better maintained at 20 years.

Attention has been given to tumour size and prognosis (Fig. 2). Now the influence of size on survival can be considered in the light of tumour grade (Fig. 10). Information regarding both size and grade was available in 989 cases followed for 5 years. It is clear that outcome is generally dependent upon size, and

FIG. 10.—Tumour size, grade and survival.

that this is greatly influenced by histological grade. Thus the prognosis for grade I cases remains good whether the tumour is small or of moderate size : it is only with the largest tumours in this grade that the outlook deteriorates appreciably. The survival rate for grade III cases is considerably lower than that for grade I cases and the influence of size is rather more marked. The outlook for patients with tumours of intermediate malignancy (grade II) occupies an intermediate position and shows the greatest change in survival with increasing tumour size.

The histological grade reflects the potential malignancy of breast cancer, and indicates which cases are more likely to have occult blood-borne metastases at the time of treatment. Metastases, however, appear to be common to all three grades of tumour when patients are first seen, and in such cases grading provides a guide to the speed with which the deposits are likely to become active, produce symptoms and cause death (Bloom and Richardson, 1957).

*Grade incidence and delay*

With increasing duration of symptoms the number of patients with grade III tumours diminishes, whilst the proportion of essentially more benign grade I cases increases. The incidence of these grades is approximately equal when the delay is 6 to 12 months (Table XVII and Fig. 11). Of the patients presenting within 3

TABLE XVII.—*Grade Incidence According to Delay* (1263 *Cases*)

| Delay | Grade I (%) | Grade II (%) | Grade III (%) | Total | (%) |
|---|---|---|---|---|---|
| ⩽ 3 months | 23 | 45 | 32 | 571 | 100 |
| >3⩽ 6 „ | 19 | 49 | 32 | 235 | 100 |
| >6⩽12 „ | 29 | 41 | 30 | 232 | 100 |
| 1–2 years | 35 | 41 | 24 | 123 | 100 |
| 2–3 „ | 33 | 46 | 21 | 39 | 100 |
| >3 „ | 41 | 51 | 8 | 63 | 100 |

FIG. 11.—Grade incidence according to delay (1263 cases)

months 23 per cent had grade I tumours and 32 per cent were grade III, compared with 41 per cent and 8 per cent respectively for patients waiting more than 3 years. The proportion of intermediate grade cases at the different periods of delay showed little change.

Histological grade appears to be a neglected factor which may alter the expected prognosis in breast cancer. Thus the promising outlook for a patient who seeks treatment promptly with a small primary tumour, or a stage 1 or stage 2 lesion, may be modified unfavourably if the grade is high. Conversely, life may be prolonged to a surprising extent in patients who present after a delay of 2 or even 3 years with locally advanced tumours of low histological grade.

*Delay and survival according to grade*

In view of the above observations the influence of delay on survival was re-examined in the light of tumour grade (Fig. 12). Initially, the survival rate is seen to decrease for patients with grade I and also grade II tumours. After a delay of between 12 and 24 months the survival rate for grade I cases recovers to become comparable to that for cases attending within 3 months. In grade II cases there is little change in survival rate after 6 months. The poorest prognosis is seen for patients with grade III tumours, and there appears to be no gain for those who seek treatment early with this highly malignant type of lesion. Although the differences are not marked, this is the first positive direct correlation between delay

Fig. 12.—Delay and prognosis according to grade (1263 cases); 5 year survival.

and survival found in the present series, at least for grade I and perhaps grade II cases. A comparable survival pattern according to tumour grade and delay was seen in 615 cases at the Royal Marsden Hospital (Smithers, 1958).

### *Combined Factors for Assessing Influence of Delay*

A more accurate assessment of the effect of delay on prognosis in breast cancer may be achieved by combining some of the individual factors that have been discussed. The subject now becomes more complex since many permutations are possible using the size, stage and grade of the primary tumour, and the state of the axillary lymph nodes for correlation with delay.

*Delay and survival according to tumour size and grade*

First, let us consider size and grade of primary tumour, in relation to patient delay and survival. Adequate information for this study was available in 883 cases (Fig. 13). It is clear that the patients coming within 3 months constitute a heterogenous group of cases with small and large tumours of different grades of malignancy and with a 5-year survival rate ranging from 87 down to 30 per cent. A similar state of affairs exists for the other periods of delay shown in Fig. 13.

In spite of some discrepancies, the prognosis for specific groups according to grade tends to deteriorate with increasing duration of symptoms (Fig. 13). Thus

FIG. 13.—Prognosis according to delay, grade and size.

when the cases seeking treatment within 3 months are compared with those coming between 3 and 12 months, the survival rate is seen to fall for patients with tumours of less than one inch in diameter, whether the lesions, during such an interval, tend to remain small or increase in size. A similar change in prognosis occurs for the larger tumours, except that grade III cases appear to have a comparable survival whether they are seen within 3 months or between 3 and 12 months. Since spontaneous regression is exceptionally rare in breast cancer the cases with large tumours are not to be compared with those coming after a longer interval with small tumours (Fig. 13). The influence of duration of symptoms on survival according to tumour grade and size is not upheld on passing from the intermediate period of delay (3–12 months) to one exceeding 12 months. It is noteworthy that the outlook for women with large tumours of grade I malignancy who delay for 3

to 12 months or longer, is better than that for patients who present within 3 months with small grade III lesions.

*Delay according to stage and grade*

The staging of breast cancer provides a valuable method of expressing the extent of the disease and brings together various local tumour factors and the condition of the regional lymph nodes which, so far, have only been used individually to investigate the influence of delay in this disease. A simple combination of stage and grade may, therefore, give the most comprehensive account of the disease possible at the present time, being a measure of the potential malignancy of the tumour and its obvious extent when first seen.

Stage of disease has already been related to delay (Table XIII ; Fig. 4 ; Table XIV). A more accurate correlation between tumour advancement and delay can be achieved when cases are considered according to both grade and stage. For each grade of tumour the time within which 25, 50 and 75 per cent of the cases attended for treatment is seen in Table XVIII. With passing time each grade advances in stage, and the higher the grade the faster the tumour's progress. For example, in grade I malignancy 75 per cent of stage I cases seek advice within 10·1 months, compared with 18 months for the same proportion of stage 3 cases. The corresponding figures for grade II cases is 6·8 and 15 months, and for grade III cases, 5·5 and 12 months.

TABLE XVIII.—*Delay According to Stage and Grade*

| Grade | Stage 1 | | Stage 2 | | Stage 3 | | Stage 4 | |
|---|---|---|---|---|---|---|---|---|
| I | (147) | 1·3* | (111) | 1·7 | (56) | 2·5 | (9) | Indeterminate |
| | | 3·3† | | 4·6 | | 8·3 | | |
| | | 10·1‡ | | 11·9 | | 18·0 | | |
| II | (188) | 1·1 | (222) | 1·1 | (119) | 2·9 | (35) | 1·9 |
| | | 3·0 | | 3·0 | | 6·4 | | 3·8 |
| | | 6·8 | | 7·3 | | 15·0 | | 15·0 |
| III | (84) | 1·3 | (162) | 1·3 | (92) | 1·4 | (30) | 3·1 |
| | | 2·5 | | 2·5 | | 4·6 | | 6·5 |
| | | 5·5 | | 5·6 | | 12·0 | | 13·0 |

() = Number of cases

* Lower quartile : Time (months) within which 25% of Cases attended
† Median : ,, ,, ,, ,, 50% ,, ,, ,,
‡ Upper quartile : ,, ,, ,, ,, 75% ,, ,, ,,

*Distribution of early and late stage cases according to grade and delay*

It has already been shown that with increasing delay the proportion of cases with tumours of limited stage falls whilst the incidence of the more advanced stages increases (Fig. 4 ; Table XIV). This relationship can now be re-examined in the light of tumour grade (Fig. 14). With increasing delay in grade I cases, the trend is for stage 1 tumours to become fewer in number whilst those in stage 3 become greater. After a delay of more than 2 years the proportion in stage 3 exceeds that in stage 1. The corresponding " cross-over " in stage distribution for grade II tumours is seen to take place just after 12 months, and for grade III

FIG. 14.—Incidence of stage 1 and stage 3 cases according to grade and delay.

tumours between 6 and 9 months. Once again cancer of the breast is shown to advance with increasing duration of symptoms, and the higher the grade of tumour the quicker the progress of the disease.

*Delay and survival according to grade and stage*

By combining clinical stage and histological grade a more accurate guide to prognosis in breast cancer can be obtained than by either system alone (Bloom, 1950*a*, 1958). Within the framework of this combined classification the influence of delay is to be found, and the results of its application support the view that survival is adversely affected by increasing the delay (Fig. 15). With advancing

FIG. 15.—Survival of grade I and grade III cases according to stage. Incidence of cases with short and prolonged delay.

stage the survival rate is seen to fall steeply for grade III cases and more gradually for grade I cases. The proportion of patients attending within 3 months and after 12 months is also shown for each group by the shaded columns. In both the grade I and the grade III cases it is evident that with advancing stage and a falling survival rate the patients with a short delay decrease in number whilst those with a prolonged delay increase. A similar picture was seen for grade II cases, the survival of which occupied an intermediate position to that of the other two grades.

*Stage and grade for prognosis*

In using the combined classification of stage and grade for breast cancer it is perhaps more convenient to group cases, as in previous communications (Bloom, 1950*a*, 1958), with sub-divisions according to grade (Fig. 16). It is clear that each stage is composed of patients with tumours of widely differing malignant potential, emphasising the inadequacy of considering cases by stage alone, especially when comparing treatment results. The survival rate within stage 2, for example, varies from as high as 71 per cent for women with grade I tumours to as low as 26 per cent for those with grade III lesions. An important feature of this classification is the revelation that patients with grade I breast cancer in the later stages of the disease have a considerably better prognosis than do less advanced cases with grade II or grade III tumours. Thus, the survival rate for stage 3 grade I cases is comparable to that for stage 1 grade II, or stage 1 grade III cases. Note that even stage 4 cases with grade I lesions have as good an outlook as the grade III

FIG. 16.—Survival by stage and grade ; 5-year results in 1393 cases.

cases in stage 2. A pattern of survival similar to that at 5 years is seen in the 10- and 15-year results, and to a lesser extent at 20 years (Fig. 17, 18, 19).

The application of this combined classification to patients with breast cancer presenting for treatment within, say, 3 months and also after 12 months from the onset of symptoms (Fig. 20 and 21), clearly emphasises the tremendous variation in the type of case seeking early or even late advice. It highlights the futility of trying to study the influence of delay, as well as other problems, in this disease by single factors alone and without reference to inherent tumour malignancy.

### DISCUSSION

It is not possible to determine the influence of delay on prognosis in breast cancer without taking into account the intrinsic biological nature of the tumour. The slow growing tumours of low grade malignancy must be separated from those of rapid growth and high malignancy. The reason that some patients who delay seeking treatment for long periods have a surprisingly high survival rate is that they represent a selected group of cases with a favourable prognosis based on the slow

**FIG. 17.—Survival by stage and grade ; 10-year results in 1341 cases.**

**FIG. 18.—Survival by stage and grade ; 15-year results in 1054 cases.**

FIG. 19.—Survival by stage and grade ; 20-year results in 458 cases.

FIG. 20.—Survival of patients with ⩽ 3 months' delay according to stage and grade progress of their tumours, or a high degree of host resistance, or both. As Berkson (1962) has pointed out, they are the survivors of a larger group of cases in which those with the more malignant tumours and poorest resistance have already been eliminated by death.

One should also add that the influence of delay is often studied in patients treated by radical surgery which automatically excludes many cases which have become advanced and inoperable because of prolonged delay. McWhirter (1957) found that patients with operable and locally advanced carcinoma of the breast who delay for one year or more had a slightly higher survival rate than those

FIG. 21.—Survival of patients with >12 months' delay according to stage and grade.

who seek treatment within 3 months. When the cases with distant metastases were included in the analysis a better prognosis was seen for patients who presented early, the incidence of distant metastases being much greater in those who delayed for a year or more. In a later report (McWhirter, 1960), covering 1985 cases, the 5-year survival rate for operable cases presenting within 2 months (59 per cent) was virtually identical to that for patients delaying for one year or more (60 per cent). When the patients with operable and inoperable tumours were considered together the survival rate for cases with the shorter history was 52 per cent compared with 39 per cent for those with symptoms extending over one year or more. A higher survival rate for women treated early was also seen in the series of over 10,000 unselected cases of breast cancer reported by the Registrar-General (1952). Of 363 cases presenting within one month 44 per cent survived 5 years, compared with 29 per cent of 487 cases who delayed for 5 to 6 months (Table XIX).

TABLE XIX.—*Survival According to Delay* (10,025 *Cases* 1945–47)

| | Delay (months) | | | | | | | | | | | |
|---|---|---|---|---|---|---|---|---|---|---|---|---|
| | 0– | 1– | 2– | 3– | 4– | 5– | 6– | 9– | 12– | 18– | 24+ | Total |
| Cases . . | 363 | 1075 | 1095 | 896 | 656 | 487 | 1287 | 483 | 1146 | 312 | 1601 | 10025 |
| Crude survival % | 44 | 42 | 39 | 35 | 35 | 29 | 29 | 31 | 27 | 31 | 27 | 33 |

Registrar-General (1952)

The necessity of having histological sections for grading purposes in the present series produced a bias in favour of operable cases, the tumours in 73 per cent being in stages 1 and 2 : 21 per cent of the cases were stage 3, and 6 per cent were stage 4. Delay *per se* was found to have little influence on survival in these cases and it was only by an indirect approach to the problem that prognosis was shown to deteriorate with increasing duration of symptoms.

Clinical stage takes into account the size of the primary tumour and the features of its local advancement in the breast, together with the condition of the axillary nodes, all of which can be correlated individually with delay and with survival. By considering histological grade we also have a direct measure of the intrinsic malignancy of the tumour—a guide to the tempo of the disease. It was shown that as each grade of tumour advances in stage with increasing delay, so the survival rate falls.

The fact that certain potentially highly malignant grade III tumours appear to be still confined to stage 1 when first seen, and carry 5-, 10- and 15-year crude survival rates of approximately 70, 50 and 40 per cent, respectively, is perhaps encouraging for those who strive to reduce delay in the hope of improving results. What is likely to be gained by such efforts?

In more recent times duration of symptoms in breast cancer has become shorter and more patients are being seen before the breast lesion has developed clinical features of frank malignancy. Of 1000 cases of mammary cancer at the Memorial Hospital 21 per cent belonged to this category (Urban, 1960). The 5-year survival rate for this group was 74 per cent compared with 52 per cent for those in whom a clinical diagnosis of malignancy was made. Axillary node involvement was present in only 32 per cent of the " early " cases compared with 60 per cent of the remainder. The better prognosis for cases without obvious clinical manifestations of malignancy was also seen in patients with axillary involvement by " high grade " tumours (Urban, 1956).

Berger *et al.* (1963) found 110 cases of unsuspected breast carcinoma in 4688 patients studied by mammography. In 28 cases the tumour was impalpable. Only 30 per cent of the 110 cases had axillary metastases. Gilbertson and Wangensteen (1963) have recently reported preliminary results from the Cancer Detection Service of the University of Minnesota. Of 25 cases with asymptomatic breast cancer treated by radical mastectomy 22 survived 5 years (88 per cent). Only 6 cases had axillary metastases (25 per cent). There was only one death in 5 years among 19 patients in whom the axilla was free (95 per cent survival).

It would seem that the detection of very early clinical and even sub-clinical breast cancer is possible before metastases have taken place, or at a time when such metastases can be overcome by host resistance. The preliminary results from cancer detection clinics are promising, but a prolonged follow-up of 15 years or more will be required before one is able to say that these cases have probably been cured. It is too early to know whether the efforts required to seek out such cases are practical or not. For example, Witten and Thurber (1964), using routine mammography, were able to discover only 8 pre-clinical carcinomas among over 5000 women, 40 years of age or older, who had no significant breast complaints or findings. The fact that it is possible in practice to detect a small number of patients with early cancer of the breast and that in such cases treatment results appear to be improved, is employed here solely to support the plea for trying to reduce delay generally in this disease—the arguments, so far, having been based entirely on retrospective studies.

Even in advanced cases delay has been shown to influence prognosis appreciably. Thus, in patients with involvement of apical axillary lymph nodes treated by radical mastectomy at the Memorial Hospital, Robbins and Bross (1957) reported that 36 per cent of patients who presented within 2 months were alive at 5 years, compared with only 19 per cent of those who delayed for more than 6 months (Table XX). A well-marked direct correlation between delay and survival, however, especially in selected material, has not been the experience of most investigators in this field.

TABLE XX.—"*Level* 3" *Axillary Involvement and Prognosis*

| Delay (months) | Cases | 5-Year survivals | (%) |
|---|---|---|---|
| <2 | 156 | 56 | 36 |
| 2–6 | 100 | 28 | 28 |
| >6 | 160 | 30 | 19 |
| Total | 416 | 114 | 27 |

From total of 1281 operable cases after Robbins and Bross (1957)

It is unlikely that prolongation of life by earlier diagnosis in breast cancer is merely due to bringing forward the date of treatment on what is, in point of fact, a fixed survival scale. Over 60 per cent of the total patients in the present series attended hospital within 6 months, and over 80 per cent within 12 months, and prognosis has been measured in terms of 5, 10 and 15 years or more.

*Intrinsic tumour and host changes with time?*

Certain further arguments may be mentioned here in favour of reducing delay in breast cancer but, at the present time, these are largely speculative. With increasing duration of symptoms changes in the tumour-host relationship might occur which affect prognosis adversely. Thus, progression in the tumour to a less responsive state is possible ; that is, less responsive to treatment by irradiation or by endocrine methods. For example, does prophylactic castration, which appears to improve results in certain groups of patients, inhibit or even destroy occult deposits and, with increasing delay, is the response of such deposits reduced or lost? During the period of waiting an alteration in hormonal balance may take place in the patient which may affect the tumour adversely. Thus, endocrine changes during pregnancy may be responsible for determining the character of a co-incident breast cancer which is generally of grade III type, and carries a particularly poor prognosis (Bloom, 1962). With increasing delay does response to irradiation alter, if not by virtue of a change in intrinsic properties, simply by an increase in tumour volume with the development of poorly oxygenated areas?

Can tumour grade alter with time, and during the period of delay progress to a more malignant type? Occasionally de-differentiation seems to take place in established tumours such as in certain cerebral gliomas. A recurrent astrocytoma, for example, may be of higher grade than the original lesion excised several years previously. On the other hand, grade of malignancy in breast cancer appears to be remarkably stable, the histological appearance of metastases presenting 10 or more years after radical mastectomy closely resembling that of the primary tumour (Bloom and Richardson, 1957).

A well-marked lymphocytic and plasma cell reaction in certain well-circumscribed breast tumours of apparently high grade malignancy (" medullary carcinoma ") is associated with a good prognosis (Moore and Foote, 1949 ; Richardson, 1956), and may reflect a high degree of host resistance to the tumour. Further support for this concept has been presented by Hultborn and Törnberg (1960) and by Berg (1959, 1962) who also find an improved outlook for patients with breast cancer associated with round cell infiltration. Another factor which may be a measure of host reaction to breast cancer and of value in prognosis is the degree of sinus hyperplasia in the axillary lymph nodes (Black *et al.*, 1955, 1956 ; Black and Speer, 1958 ; Wartmann, 1959 ; DiRe and Lane, 1963), although Berg (1956) and also Moore *et al.* (1960) have been unable to confirm this. It is interesting to consider whether these reactions, claimed to represent host resistance, may be modified unfavourably during a period of delay by, for example, endocrine changes, local infection or intercurrent disease.

In his monograph on the significance of delay in cancer in general, Sutherland (1960) deals at some length with host resistance factors and this subject has also been reviewed recently by Southam (1961). Until the above questions can be answered it would seem reasonable to strive for prompt treatment in breast cancer, not only to deal with the tumour at an early stage, but also to reduce the chance of deleterious changes taking place with time in the tumour-host relationship.

McKinnon's (1955) view that many stage 1 breast tumours diagnosed as carcinoma, in patients who subsequently do well following treatment, are not really malignant is quite untenable. All cases in the present series, including the grade I cases, were examples of histologically confirmed invasive carcinoma. On the other hand, it may be argued that good results from treatment of breast cancer are mainly found in patients with what appear to be naturally favourable tumours. Although this is undoubtedly correct it should not be employed to decry the value of treatment in general since breast cancer, even of low grade malignancy, does advance, metastasize and eventually kill the host if untreated. Furthermore, even in those patients where there is the suggestion of a high natural resistance, such as cases of " medullary carcinoma ", death will occur from widespread disease if therapy is inadequate (Richardson, 1956).

Fifteen years ago the present author expressed the view that outcome in mammary cancer was largely determined by the biological character of the tumour, reflected in the histological grade, rather than by prompt treatment (Bloom, 1950*b*). Macdonald (1942) has voiced a similar opinion over a longer period. Although in the main this view must still be upheld it nevertheless appears that delay may lose years of life and comfort for many patients, especially for those with tumours of intermediate and of low grade malignancy. It is perhaps just those women who delay for long periods with tumours of relatively low grade malignancy, or with a high natural resistance, who may have a better prognosis or chance of cure if they were treated at an earlier date. Radical treatment in a particular case might have been feasible some months previously whereas now only palliation is possible.

With the passing years an improvement in the results of breast cancer treatment has been reported by a number of observers (Adair, 1949; Taylor, 1949, Harrington, 1952; Haagensen, 1956). The average delay in seeking medical advice after onset of symptoms also appears to be shorter (Harrington, 1946; Leach and Robbins, 1947; Nohrman, 1949; Moore and Shaw, 1957). At the same time the proportion of patients presenting with smaller tumours, negative axillary nodes, limited node involvement, or in stage 1 of the disease, is greater (Moore and Shaw, 1957; Berkson *et al.*, 1957; Robbins *et al.*, 1959; McSwain and Fleming, 1963). These changes are considered to be responsible for the improvement in survival rate seen in recent years in some centres (Moore and Shaw, 1957; Berkson *et al.*, 1957). At the Mayo Clinic the increase in survivals is confined to patients with positive axillary nodes (Berkson *et al.*, 1957). This is probably also due to the treatment of earlier and less advanced cases, prognosis being not only related to the presence or absence of axillary metastases, but also to the number and level of nodes involved.

Unlike the vague symptoms so often associated with the clinical onset of malignant disease involving internal organs, cancer of the breast provides the patient with objective, tangible evidence of its existence—a lump, which is the first symptom in over 80 per cent of cases. In spite of the present-day education and publicity on medical matters received by the general public, there is still in many places a distressingly high proportion of women who delay seeking advice for a breast tumour. Of 943 patients seen between the years 1955 and 1959 in London at the Royal Marsden Hospital no less than 50 per cent presented with a primary tumour greater than 5 cm., or with skin ulceration (Harmer, 1962). It is difficult to believe that in these circumstances the best possible prognosis is being secured for patients with breast cancer.

In previous communications (Bloom *et al.*, 1962; Bloom, 1964) the benefit to be derived from treatment *per se* in breast cancer was emphasised by considering the natural history of the untreated disease. In the present paper the effects of delay on the tumour and perhaps the host have been reviewed and now a plea is made for early diagnosis and prompt treatment. Before closing, however, it is important to stress that, since half the patients who appear to come early for treatment already have axillary node involvement, the overall improvement in survival resulting from efforts to reduce delay alone is likely to be limited. Before we can materially alter the outlook for many cases we may have to wait for new methods of treatment. On the other hand, Robbins and Bross (1957) were able to demonstrate a profound effect of delay even in advanced cases who were treated by standard radical surgery (Table XX). We surely cannot avoid striving to reduce the delay period in all patients for the sake of the limited number whose life may thereby be saved, and for a greater number of others who are likely to gain time and comfort: because of the possible benefit for the individual which can be so easily overlooked in mass statistics, early treatment must be the undoubted principle for all cases.

SUMMARY

Most authors are unable to demonstrate an appreciable deterioration in prognosis in breast cancer with increasing duration of symptoms. In the present series of over 1200 cases treated chiefly by radical mastectomy with or without ancillary irradiation, delay in treatment did not appear to influence prognosis adversely, as judged by the 5- to 20-year survival rates. The outlook for patients presenting at hospital within 3 months was identical to that for cases coming after a delay of one year or more. It was only when attention was given to intrinsic tumour malignancy and to the influence of lost time on the tumour itself, that the harmful effect of delay in treatment could be demonstrated.

Between the onset of symptoms and the institution of treatment breast cancer, as judged by individual tumour factors, clearly tends to develop more unfavourable characteristics. With increasing delay smaller tumours are found less frequently and larger tumours more often. As time passes not only do more cases develop axillary metastases, but the extent of this involvement increases, as judged by the number and level of nodes invaded. The larger the primary tumour the higher the incidence of axillary deposits. The greater the degree of axillary involvement the greater the risk of spread to other regional nodes. As delay increases fewer patients with low stage tumours and more with high stage lesions are seen : the proportion of inoperable cases becomes greater. With advancing stage the median and upper quartile periods of delay become longer.

Since all the above tumour factors can be shown to advance with increasing duration of symptoms, and since increasing changes in the tumour are associated with a falling survival rate, delay itself must affect prognosis adversely.

A reason for the failure to demonstrate a direct correlation between delay and survival became evident after taking into account the intrinsic malignancy of the tumour, based on histological grade. The more rapidly growing and more lethal grade III tumours tend especially to be found in patients who seek early treatment, presumably because this type of tumour produces more alarming symptoms. On the other hand, the essentially less malignant grade I lesions are found more often in patients with a long history. The distribution of tumour grades tends to counterbalance the influence of delay on survival rate.

By employing histological grade in the study of breast cancer the broad spectrum of malignancy which exists in any group of patients with this disease is more fully appreciated. Those patients seeking advice within, for example, 3 months, are composed of women with small and large tumours of different grades of malignancy, and with a 5-year survival rate ranging from 87 per cent to 30 per cent.

The effect of delay on various individual tumour factors was re-examined in the light of tumour grade. Since clinical stage takes into account many of the local features of the breast lesion, together with the state of the axillary nodes, the simplest and most comprehensive account of mammary cancer appears to be given by a combination of histological grade and clinical stage. This provides a guide to the intrinsic properties of the tumour, including the tempo of the disease, and is a measure of the obvious extent of the lesion when first seen.

As each grade of tumour advances in stage so the median and upper quartile periods of delay increase. The higher the grade, the quicker this advance takes place.

With increasing delay for each grade of tumour, the incidence of stage 1 cases decreases, whilst that of stage 3 cases becomes greater. The relative proportion of these stages at any given time depends upon tumour grade, the higher the grade the sooner the more advanced cases predominate.

The survival rate falls markedly with increasing stage, and the extent of this deterioration depends upon tumour grade. A combined classification using stage and grade provides a more accurate guide to prognosis than either factor alone, and within the framework of this classification the influence of delay can be seen.

By means of the combined classification it was possible to show that tumours of all stages and grades are represented among cases seeking early advice (within 3 months). Such cases had a 5-year survival rate ranging from as high as 88 per cent for women with stage 1, grade I carcinomas to only 14 per cent for those with stage 3, grade III lesions. Within the limits of a single stage, stage 2, the survival rate for patients attending hospital within 3 months with grade I tumours was 84 per cent, compared with only 24 per cent for those with grade III lesions. An equally striking variety of cases was found even among women harbouring breast tumours for more than a year. It is, therefore, impossible to assess the influence of delay on survival in breast cancer without a classification which takes into account the wide variation in type of case met with at all times in this disease.

It was speculated that certain intrinsic factors such as grade, hormonal responsiveness and radiosensitivity of the tumour, and possibly resistance of the host, might be modified unfavourably during the interval between a patient finding a breast lump and seeking advice.

Since almost half the patients with breast cancer who appear to seek treatment early already have axillary metastases, the overall improvement in survival rate resulting from efforts to reduce delay is likely to be limited. On the other hand, since half the patients with this disease attending a cancer hospital in a large city seek advice only when their tumours are greater than 5 cm., or ulcerated, it is difficult to believe that present day methods of treatment are achieving the best possible results. Patients who come late for treatment often have tumours of low grade malignancy, and it may be just these cases that will benefit most from efforts to reduce delay.

In a previous communication the author and his colleagues (Bloom, Richardson and Harries, 1962) emphasised the importance of treatment *per se* in breast cancer by considering the natural history of the untreated disease. In the present paper the effects of delay in treatment on the tumour and possibly also on host resistance have been reviewed, and a plea is made for efforts to reduce loss of time. Because of the chance of saving or prolonging useful life for the *individual*, a factor often overlooked in mass statistics, early treatment must be the undoubted principle for all cases of breast cancer.

I am grateful to the medical staff of the Middlesex Hospital for the cases employed in this study and to Mr. T. E. Cowan of the Records Department for follow-up data. I remain indebted to Professor R. W. Scarff of the Bland-Sutton Institute for introducing me to the subject of tumour grading. The histological sections of two-thirds of the cases in this series were formerly studied in collaboration with Mr. W. W. Richardson and were part of the material for our joint paper on grading of breast cancer.

My thanks are due to Mr. P. Payne, Director of the S.E. Metropolitan Cancer Registry, for his advice and help in assembling the statistical data.

I should also like to thank the Departments of Medical Art and Photography of the Royal Marsden Hospital for the figures. Fig 7 and 8 have been reproduced by kind permission of the New York Academy of Sciences.

### REFERENCES

Ackerman. L, V.—(1952) *Proc. 2nd nat. Cancer Conf., New York*, **1**, 194.
Adair, F. E.—(1949) *Ann. R. Coll. Surg. Engl.*, **4**, 360.
Andreassen, M., Dahl-Iverson, E. and Sørensen, B.—(1954) *Lancet*, i, 176.
Berg, J. W.—(1955) *Cancer*, **8**, 776.—(1956) *Ibid.*, **9**, 935.—(1959) *Ibid.*, **12**, 714.—(1962) *Acta Un. int. Cancr.***18**, 854.
Berger, S. M., Gershon-Cohen, J. and Behrend, A.—(1963) *Arch. Surg.*, **86**, 308.
Berkson, J.—(1962) *Acta Un. int. Cancr.*, **18**, 1003.
*Idem*, Harrington, S. W., Clagett, O. T., Kirklin, J. W., Dockerty, M. B. and McDonald, J. R.—(1957) *Proc. Mayo Clin.*, **32**, 645.
Black, M. M., Opler, S. R. and Speer, F. D.—(1955) *Surg. Gynec. Obstet.*, **100**, 543.
*Idem* and Speer, F. D.—(1958) *Ibid.*, **106**, 163.
*Iidem* and Opler, S. R.—(1956) *Amer. J. clin. Path.*, **26**, 250.
Bloom, H. J. G.—(1950*a*) *Brit. J. Cancer*, **4**, 259.—(1950*b*) *Ibid.*, **4**, 347.—(1958) *Proc. R. Soc. Med.*, **51**, 122.—(1962) *Acta Un. int. Cancr.*, **18**, 842.—(1964) *Ann. N.Y. Acad. Sci.*, **114**, 747.
*Idem* and Richardson, W. W.—(1957) *Brit. J. Cancer*, **11**, 359.
*Iidem* and Harries, E. J.—(1962) *Brit. med. J.*, ii, 213.
Budinger, J. M.—(1964) *Radiology*, **83**, 255.
Burdick, D. and Chanatry, F.—(1954) *Cancer*, **7**, 47.
Collins, V. P., Loeffler, K. and Tivey, H.—(1956) *Amer. J. Roentgenol.*, **76**, 988.
Dahl-Iversen, E.—(1956) *Proc. 3rd nat. Cancer Conf., Detroit*, p. 148.
Delario, A. J.—(1960) ' Breast Cancer. Factors Modifying Prognosis '. New York (Macmillan) p. 123.
DiRe, J. J. and Lane, N.—(1963) *Amer. J. clin. Path.*, **40**, 508.
Eggers, C., de Cholnoky, T. and Jessup, D. S. D.—(1941) *Ann. Surg.*, **113**, 321.
Eker, R., Stokke, T. and Efskind, J.—(1958) *Saertrykk* fra 25th Anniversary Publcn. from Norwegian Radium Hospital, p. 173.
Foulds, L.—(1951) *Ann. R. Coll. Surg.*, **9**, 93.
Geschickter, C. F.—(1945) ' Diseases of the Breast '. Philadelphia (J. B. Lippincott and Co.) pp. 410D, 460, 592.
Giacomelli, V. and Veronesi, U.—(1952) *Tumori*, **38**, 375.
Gilbertson, V. A. and Wangensteen, O. H.—(1963) *Surg. Gynec. Obstet.*, **116**, 413.
Greenough, R. B.—(1925) *J. Cancer Res.*, **9**, 453.
Haagensen, C. D.—(1956) ' Diseases of the Breast '. Philadelphia, (W. B. Saunders and Co.) pp. 385, 424, 636.
*Idem* and Stout, A. P.—(1942) *Ann. Surg.*, **116**, 801.—(1951) *Ibid.*, **134**, 151.
Halsted, W. C.—(1895) *Johns Hopk. Hosp. Rep.*, **4**, 297.
Handley, R. S.—(1962) *Acta Un. int. Cancr.*, **18**, 876.
*Idem* and Thackray, A. C.—(1954) *Brit. med. J.*, i, 61.
Harmer, M.—(1962) *Acta Un. int. Cancr.*, **18**, 982.
Harnett, W. L.—(1953) *Brit. J. Cancer* **7**, 19.
Harrington, S. W.—(1946) *Surgery*, **19**, 154.—(1952) *J. Amer. med. Ass.*, **148**, 1007.
Hoopes, B. F. and McGraw, A. B.—(1942) *Surgery*, **12**, 892.
Hultborn, K. A. and Törnberg, B.—(1960) *Acta radiol., Stockh.*, Suppl., 196.
Kaae, S.—(1948) *Ibid.*, **29**, 475.
Lalanne, C. M.—(1962) *Acta Un. int. Cancr.*, **18**, 807.
Lane, N., Goksel, H., Salerno, R. A. and Haagensen, C. D.—(1961) *Ann. Surg.*, **153**, 483.

Leach, J. E. and Robbins, G. F.—(1947) *J. Amer. med. Ass.*, **135**, 5.
Luff, A. P.—(1932) *Brit. med. J.*, i, 897.
Margottini, M.—(1948) *Oncology*, **22**, 281.
Macdonald, I.—(1942) *Surg. Gynec. Obstet.*, **74**, 75.
McKinnon, N. E.—(1951*a*) *Canad. J. publ. Hlth.*, **42**, 218.—(1951*b*) *Ibid.*, **42**, 88.—(1954) *Lancet*, i, 251.—(1955) *Canad. med. Ass. J.*, **73**, 614.
McSwain, B. and Fleming, J. H.—(1963) *Cancer*, **16**, 681.
McWhirter, R.—(1957) *J. Fac. Radiol., Lond.*, **8**, 220.—(1960) *Clin. Radiol.*, **11**, 144.
Moore, C. and Shaw, H. W.—(1957) *Arch. Surg.*, **75**, 598.
Moore, C. S. and Foot, F. W.—(1949) *Cancer*, **2**, 635.
Moore, R. D., Chopnick, R. and Schoenberg, M. D.—(1960) *Ibid.*, **13**, 545.
Nohrman, B. A.—(1949) *Acta radiol., Stock.*, Suppl., **77**, 36.
Park, W. W. and Lees, J. C.—(1951) *Surg. Gynec. Obstet.*, **93**, 129.
Paterson, R.—(1948) ‘ Treatment of Malignant Disease by Radium and X-rays ’, London (Arnold) p. 309.
Patey, D. H. and Scarff, R. W.—(1928) *Lancet*, i, 801.
Registrar-General—(1952) Statistical Review of England and Wales, Supplement on Cancer, London (H.M. Stationery Office) p. 9.
Rennaes, S. (1960) *Acta chir. scand.*, Suppl., 266.
Richards, G. E. (1948) *Brit. J. Radiol.*, **21**, 109.
Richardson, W. W. —(1956) *Brit. J. Cancer*, **10**, 415.
Rigby-Jones, P.—(1962) *Acta Un. int. Cancr.*, **18**, 815.
Robbins, G. F.—(1962) *Ibid.*, **18**, 864.
*Idem* and Bross, I.—(1957) *Cancer*, **10**, 338.
*Idem*, Berg, J. W., Bross, I., de Padua, C. and Sarmiento, A. P.—(1959) *Ibid.*, **12**, 688.
Schwartz, M.—(1961) *Ibid.*, **14**, 1272.
Shimkin, M. B., Eschecholtzia, L. L., Stone, R. S. and Bell, H. G.—(1952) *Surg. Gynec. Obstet.*, **94**, 645.
Smithers, D. W.—(1958) *Amer. J. Roentgenol.*, **80**, 740.
Southam, C. M.—(1961) *Med. Clin. N. Amer.*, **45**, 733.
Sutherland, R.—(1960) ‘ Cancer. The Significance of Delay ’, London (Butterworth and Co.) p. 64.
Swynnerton, B. F. and Truelove, S. C.—(1952) *Brit. med. J.*, i, 287.
Taylor, G. W.—(1949) *Amer. J. Roentgenol.*, **52**, 342.
Tubiana, M.—(1964) *Clin. Radiol.*, **15**, 142.
Urban, J.—(1956) *Cancer*, **9**, 1173.—(1960) *Postgrad. Med.*, **27**, 389.
Wartman, W. B.—(1959) *Brit. J. Cancer*, **13**, 389.
Waxman, B. D. and Fitts, W. T.—(1959) *Amer. J. Surg.*, **97**, 31.
Witten, D. M. and Thurber, D. L.—(1964) *Amer. J. Roentgenol.*, **92**, 14.
Zeidman, I., McCutcheon, M. and Coman, R.—(1950) *Cancer Res.*, **10**, 357.